# Model-informed health and socio-economic benefits of enhancing global equity and access to Covid-19 vaccines

**DOI:** 10.1038/s41598-023-48465-y

**Published:** 2023-12-07

**Authors:** Matteo Italia, Fabio Della Rossa, Fabio Dercole

**Affiliations:** Department of Electronic, Information, and Bioengineering, Politecnico di Milano, Milan, Italy

**Keywords:** Infectious diseases, Ecological epidemiology, Population dynamics, Applied mathematics, Information technology, Scientific data, Engineering, Mathematics and computing

## Abstract

We take a model-informed approach to the view that a global equitable access (GEA) to Covid-19 vaccines is the key to bring this pandemic to an end. We show that the equitable redistribution (proportional to population size) of the currently available vaccines is not sufficient to stop the pandemic, whereas a 60% increase in vaccine access (the global share of vaccinated people) would have allowed the current distribution to stop the pandemic in about a year of vaccination, saving millions of people in poor countries. We then investigate the interplay between access to vaccines and their distribution among rich and poor countries, showing that the access increase to stop the pandemic gets minimized at + 32% by the equitable distribution (− 36% in rich countries and + 60% in poor ones). To estimate the socio-economic benefits of a vaccination campaign with enhanced global equity and access (eGEA), we compare calibrated simulations of the current scenario with a hypothetical, vaccination-intensive scenario that assumes high rollouts (shown however by many rich and poor countries during the 2021–2022 vaccination campaign) and an improved equity from the current 2.5:1 to a 2:1 rich/poor-ratio of the population fractions vaccinated per day. Assuming that the corresponding + 130% of vaccine production is made possible by an Intellectual Property waiver, we show that the money saved on vaccines globally by the selected eGEA scenario overcomes the 5-year profit of the rights holders in the current situation. This justifies compensation mechanisms in exchange for the necessary licensing agreements. The good news is that the benefits of this eGEA scenario are still relevant, were we ready to implement it now.

## Introduction

From January 2021 to December 2022, we have administered about 13 billion doses of vaccinations against the SARS-CoV-2 (Covid-19), an amount in principle sufficient to reach worldwide herd immunity (70–80% of the population covered^1–3^). However, only 25% of people received the first dose in low-income countries, whereas we administered the fourth dose in rich countries^4,5^. The unequal access to Covid-19 vaccines worldwide is considered the leading cause of our apparent failure against the pandemic^6,7^. As a result, we are still exposed to potentially harmful outbreaks, in a scenario that many consider unavoidably set to become endemic^8^.

To end the Covid-19 pandemic, we need to do more. We definitely need to boost up vaccine production, while keeping vaccines updated to the virus evolution and possibly developing a broadly protective “universal” vaccine (against all betacoronaviruses)^9^. At the same time, we need to make vaccine administration more equally distributed worldwide. In a few words, we need “Working for Global Equitable Access to Covid-19 vaccines” (GEA), as stated in the mission of COVAX, the WHO’s (World Health Organization) pillar project on vaccines^10^.

These needs fueled the discussion on temporarily waiving intellectual property (IP) rights on Covid-19 vaccines, a proposal put forward by India and South Africa in October 2020, later supported by around a hundred members (over 164) at WTO (World Trade Organization) members (including US, Russia, and China), but opposed by EU and UK^11,12^. The public access to vaccine patents would increase production and push prices toward production costs, allowing poor countries to acquire more doses and, at the same time, enhancing the COVAX charitable scheme. Opposers (including pharmaceutical companies holding patents) argue that IP rights incentivize the development and updating of vaccines, the necessary materials would be in short supply, and setting up new productions and passing know-how could take too long. A compromise agreement was reached in June 2022 among all WTO members: it essentially broadens the scope of compulsory licensing, an existing WTO’s agreement on IP that allows governments, under emergency, to ask third parties to produce a product without the consent of the patent owner. The agreement only concerns Covid-19 vaccines, but not diagnostics and therapeutics, compensates the rights holders, and allows developing countries to produce vaccines for 5 years.

In this work, we take a model-informed approach to investigate the benefits of GEA to Covid-19 vaccines. Defining the meaning of equitable access is a controversial issue^13^: we take the simplest approach of distributing vaccines proportionally to population size. We therefore denote as enhanced global equity and access (eGEA) any vaccination strategy that, compared to the status quo, is characterized by both an increased access to vaccines (i.e., increased total production and administration) and a more equitable distribution between countries (i.e., a distribution that better reflects countries’ populations).

Epidemiological models^14,15^ are fundamental for understanding epidemic evolution and optimal control strategies to deploy scarce resources when epidemics occur in different but interconnected regions^16–18^. Many compartmental models extending the well-known SIR (Susceptible, Infectious, Recovered) model^19^ have been proposed for the Covid-19 pandemic, e.g.,^20–24^. This modeling approach is reasonably predictive for infectious diseases that are transmitted among humans^25^. A few works specifically investigated the effect of different Covid-19 vaccine allocation strategies via a modeling approach^26,27^. In particular, Moore et al.^26^ analyzes the effect of equally redistributing the vaccine doses administrated during the year 2021, while^27^ studies the advantage of using an infection-dependent allocation strategy of the vaccines with respect to a constant one.

Here, we develop a new calibrated epidemiological model integrating all principal aspects of the Covid-19 pandemic (susceptible, exposed, presymptomatic, symptomatic and asymptomatic infected, quarantined, hospitalized, recovered, and death subjects; contact tracing; containment measures; vaccination campaign), and we simulate the pandemic dividing nations into high-income (HI) and middle-to-low-income (MLI), interconnected by mobility flows. We then investigate the effects of different vaccination strategies by varying both the global *access to vaccines* (specifically, the fraction of the world population vaccinated daily) and the *inequity in access* between HI and MLI countries. We conclude that the key to stop the pandemic is the combined effect of these two control parameters (note that only the second is investigated in^26^): both access and equity must be improved to eradicate SARS-CoV-2.

We further present in detail a hypothetical vaccination-intensive eGEA scenario that could have been realized, since characterized by vaccine rollouts in HI and MLI countries effectively achieved during the 2021–2022 vaccination campaign, and by a 2:1 rollout ratio slightly more equitable than the 2.5:1 ratio maintained so far^4,5, 28^. Our simulations envisage the pandemic ending in about a year of vaccinations, before the emergence of virus variants that compromised the vaccines’ efficacy^29–33^. In contrast, the simulation of the status quo sees the virus turning endemic, with impressing difference in terms of total cases and lost human lives, especially in MLI countries. We also estimate the stringency index, a quantification of the containment measures adopted by governments^34^, to get a measure of the socio-economic benefits of the selected eGEA scenario, that we compare with a 5-year simulation of the status quo.

## Material and methods

We divide nations into high- and middle-to-low-income (HI and MLI, respectively). According to the World Bank’s classification for 2022, the first group includes nations with per-capita GNI (gross national income) larger than 12,695 USD, representing 16% of the world population (middle-income: per-capita GNI between 1045 and 12,695 USD). Each macro-group of countries is described by an epidemiological model, and the two models are interconnected to account for inter-group people’s mobility. Our focus is not on mobility between rich and poor countries, that involves migration flows, so that, for the purpose of this study, we assume a weak symmetric interconnection to allow the spread of infections. We extend the epidemiological model proposed by Gatto et al.^21^, to describe the initial phase of the Covid-19 outbreak in Italy. Gatto et al.’s model is particularly suited to the Covid-19 epidemic because it considers presymptom transmission^35^.

**Figure 1:**
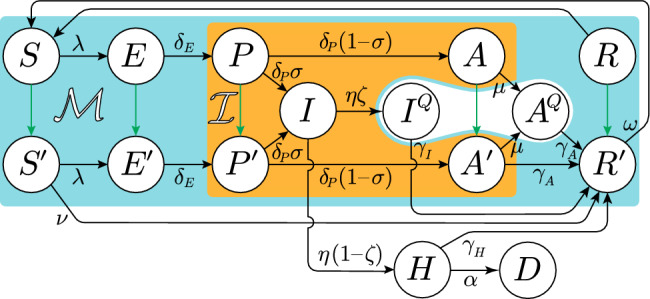
Graphical representation of the proposed epidemiological model on a single macro-group. Each node represents a possible compartment in which the group’s population is divided. Susceptible *S* ($S'$ if recently vaccinated), exposed *E* ($E'$ if recently vaccinated), presympthomatic infectious *P* ($P'$ if recently vaccinated), asympthomatic infectious *A* ($A'$ if recently vaccinated; $A^Q$ if quarantined), sympthomatic infectious *I* ($I^Q$ if quarantined), hospitalized (*H*), death (*D*), and resistant (unaware *R*, recovered from unconfirmed infections; aware $R'$, vaccinated or recovered from a confirmed infection). Arrows connecting states represent flows between the classes, with rate indicated by the parameter on the arrow. The interconnection between the macro-groups occurs through the forces of infection $\lambda$ and of contact tracing $\mu$, where citizens mobility is accounted (see Eqs. (5) and (6)). Cyan group: ${\mathcal {M}}=\{S,S',E,E',P,P',I,A,A',R,R'\}$, mobile classes; orange group: ${\mathcal {I}}=\{P,P',I,A,A'\}$, infectious classes; green arrows: vaccination flows. Tables S1 and S2 resume model classes and parameters, respectively.

Our extension is graphically represented in Fig. 1 for a single macro-group. It consists of a single-dose compulsory vaccination, in which recently vaccinated susceptibles (compartment/class $S'$) take an average of 2 weeks (transition from $S'$ to the class $R'$, parameter $\nu =1/14\,\text {day}^{-1}$^36^), before turning fully protected against infection.

A summary of the model variables, one for each compartment, and the meaning and values of the model parameters are summarized in the SI Tabs. S1 and S2. The calibration procedure for the choice of the parameters values is detailed in the SI Sect. S2^56−68^.

Note that subjects in classes exposed (*E*, infected during latency, not infectious), presymptomatic (*P*, incubation after latency, infectious), and asymptomatic (*A*, infectious without symptoms) do get vaccinated (green arrows in Fig. 1 toward classes $E'$, $P'$, and $A'$, respectively), because unaware of being infected. Vaccination is however assumed to be ineffective on already infected subjects, for which the course of the disease proceeds unaltered.

Differently from Gatto et al.^21^ and motivated by the availability of cheap diagnostic tools, strengthen average protection against severe outcomes, and improved medical intervention with respect to the early stages of the pandemic, we assume that symptomatic subjects (class *I*) are all confirmed infected (positive swabbed) in a day on average (parameter $\eta =1\,\text {day}^{-1}$) and then immediately isolated, either quarantined at home (transition from *I* to class $I^Q$) or hospitalized (to class *H*), without the possibility of recovering or dying before isolation. In reality, most symptomatic subjects are first quarantined and only later hospitalized if symptoms get worse. However, from the modeling point of view, we can separate quarantined and hospitalized subjects since from infection certification, as they are in any case assumed isolated. We also isolate asymptomatic subjects because of contact tracing (transitions from classes *A* and $A'$ to $A^Q$), an element that we believe significant in the pandemic long-term evolution. We only account for the deaths of hospitalized people (transition from *H* to *D*), but we identify from data a critical hospital occupancy threshold $H^*$ above which we increase the mortality rate (see SI Sect. S2.4), to account for deaths during home-isolation (that did occur in the early stages of the pandemic) and hospital saturation.

### Modeling protection and vaccination

We do not distinguish between the protection acquired through vaccination or while recovering from a confirmed infection (both flows—from $S'$ the former, from $I^Q$, $A^Q$, and *H* the latter—go into class $R'$). We however distinguish, in the recovery from an unconfirmed infection, between recently vaccinated and unvaccinated subjects (flows from $A'$ into $R'$ and from *A* to *R*, respectively) since the latter, being unaware of their protection, are eligible for vaccination. A vaccine dose renews the protection (transition from *R* to $R'$). In any case, the protection is assumed to wane in 6 months on average (parameter $\omega =1/180\,\text {day}^{-1}$^50,51^), after which subjects are considered again susceptibles (transition from *R* and $R'$ to *S*).

The vaccination campaign in each macro-group, once started, is assumed to proceed at a constant rollout per day $\rho$—the fraction of the group population *N* that can receive a dose of vaccine in a day. If the eligible fraction ($(S+E+P+A+R)/N$), is larger than $2\rho$, we assume that all daily available doses are successfully administered and proportionally distributed among the eligible classes. The number of doses $V^X$ administered to individuals in class $X\in \{S,E,P,A,R\}$ per day is therefore

1 $$\begin{aligned} V^X=\rho N\,\frac{X}{S+E+P+A+R}. \end{aligned}$$

Conversely, when the eligible fraction is below $2\rho$, the rollout fraction $\rho$ in Eq. (1) is reduced to half of the eligible fraction (resulting in $V^X=X/2$), to consider small density effects in tracing unvaccinated people.

The vaccination rates $\rho _1$ and $\rho _2$ in HI and MLI countries are key parameters in our model (hereafter indexes 1 and 2 are used to denote the HI and MLI macro-groups, respectively). In the current scenario, vaccination proceeded in 2021 at an average rate of $0.48\%$ in HI countries and $0.24\%$ in MLI ones (excluding the outlier China; see data and discussion in SI Sect. S2.6), so that we set $\rho _1=0.005$ and $\rho _2=0.002$. Changing their values, we can hypothetically investigate different vaccination campaigns. In particular, instead of looking at the two rates in HI and MLI countries, we equivalently focus on the total availability of vaccines worldwide and on the equity of their distribution among HI and MLI nations. We therefore define the

2 $$\begin{aligned} \text{access to vaccines} = \frac{N_1 \rho _1 + N_2 \rho _2}{W} \end{aligned}$$

as the fraction of the world population *W* vaccinated daily, where $N_1=0.16\,W$ and $N_2=0.84\,W$ are the populations of the two macro-groups, and the

3 $$\begin{aligned} \text{inequity in access} = \log _{10} \frac{\rho _1}{\rho _2}. \end{aligned}$$

Zero inequity in access represents GEA vaccination scenarios ($\rho _1\!=\!\rho _2$), whereas $\pm 1$ inequity corresponds to the two highly unbalanced situations in which the vaccination rate in one group, HI and MLI countries, respectively, is 10-times higher than in the other group.

### Modeling disease transmission

The mathematical model describing the compartmental dynamics of each macro-group of nations—HI and MLI, $i=1,2$—is the following set of ODEs:

4a $$\begin{aligned} \dot{S}_i= & {} -\lambda _i^S S_i - V_i^S + \omega (R_i+R'_i) \end{aligned}$$

4b $$\begin{aligned} \dot{S}'_i= & {} -\lambda _i^{S'} S'_i + V_i^S - \nu S'_i \end{aligned}$$

4c $$\begin{aligned} \dot{E}_i= & {} \lambda _i^S S_i - V_i^E - \delta _E E_i \end{aligned}$$

4d $$\begin{aligned} \dot{E}'_i= & {} \lambda _i^{S'} S'_i + V_i^E - \delta _E E'_i \end{aligned}$$

4e $$\begin{aligned} \dot{P}_i= & {} \delta _E E_i - V_i^P - \delta _P P_i \end{aligned}$$

4f $$\begin{aligned} \dot{P}'_i= & {} \delta _E E'_i + V_i^P - \delta _P P'_i \end{aligned}$$

4g $$\begin{aligned} \dot{I}_i= & {} \sigma \delta _P (P_i+P'_i) - \eta I_i \end{aligned}$$

4h $$\begin{aligned} \dot{I}^Q_i= & {} \zeta \eta I_i - \gamma _I I^Q_i \end{aligned}$$

4i $$\begin{aligned} \dot{A}_i= & {} (1-\sigma )\delta _P P_i - V_i^A - \mu _i^{A} A_i - \gamma _A A_i \end{aligned}$$

4j $$\begin{aligned} \dot{A}'_i= & {} (1-\sigma )\delta _P P'_i + V_i^A - \mu _i^{A'} A'_i - \gamma _A A'_i \end{aligned}$$

4k $$\begin{aligned} \dot{A}^Q_i= & {} \mu _i^{A} A_i + \mu _i^{A'} A'_i - \gamma _A A^Q_i \end{aligned}$$

4l $$\begin{aligned} \dot{H}_i= & {} (1-\zeta )\eta I_i - \gamma _H H_i - \alpha H_i \end{aligned}$$

4m $$\begin{aligned} \dot{D}_i= & {} \alpha H_i \end{aligned}$$

4n $$\begin{aligned} \dot{R}_i= & {} \gamma _A A_i - V_i^R - \omega R_i \end{aligned}$$

4o $$\begin{aligned} \dot{R}'_i= & {} \nu S'_i + V_i^R + \gamma _I I^Q_i + \gamma _A (A'_i + A^Q_i) + \gamma _H H_i - \omega R'_i \end{aligned}$$

where the sum of variables equals $N_i$ at all times ($N_1\!=\!0.16\,W$, $N_2\!=\!0.84\,W$; variables denote actual population numbers if *W* is set to the world population, otherwise fractions of word population if *W* is scaled to 1; the fractions $X_i/N_i$ of the population in class $X_i$ within group *i* are shown in the simulations).

The forces of infection $\lambda ^S_i$ and $\lambda ^{S'}_i$ are the rates at which susceptible subjects of class $S_i$ and $S'_i$ get infected (Eq. (4a–d)). Here is where people’s mobility between HI and MLI countries is taken into account, through a group-dependent force of infection that considers infections within the group and those imported from the other group (recall that only people in class ${\mathcal {M}}=\{S,S',E,E',P,P',I,A,A',R,R'\}$ are mobile—free to move within and across the two groups—the other being hospitalized or quarantined, see Fig. 1). Specifically, following Gatto and coauthors^21^, a time-independent probability $C_{ij}^{X}$ for an individual resident in a nation of group *i* and belonging to the mobile class $X\in {\mathcal {M}}$ to be present in a nation of group *j* (including $j=i$, $\sum _j C_{ij}^{X}\!=\!1$) is introduced. Then, the force of infection for a group-*i*-susceptible in class $X\in \{S,S'\}$ is defined by the sum of the so-called frequency-dependent contact rates with the infectious class $Y\in {\mathcal {I}}=\{P,P',I,A,A'\}$ in both groups $j=1,2$, each weighted by the transmission rates $\beta ^Y_j$ of the infectious class in group *j*, i.e.,

5 $$\begin{aligned} \lambda ^X_i\! = \sum _{j=1}^2 C_{ij}^X\frac{ \sum _{Y\in {\mathcal {I}} } \beta ^Y_j\sum _{k=1}^2 C_{kj}^Y Y_k}{ \sum _{Y\in {\mathcal {M}}} \sum _{k=1}^2 C_{kj}^Y Y_k}. \end{aligned}$$

The rationale behind this assumption is that the mobile classes include the majority of people, so that the fraction of non-mobile subjects has little impact on the number of daily encounters—the contact rate (per day)—that, on average, can be considered a constant model parameter. Including this parameter in the transmission rates $\beta ^Y_j$, what matters is the probability that the contact occurs with a subject in the infectious class *Y*, given by the frequency of the class in the mobile population present in group *j* (the fraction that multiplies $C_{ij}^X\beta ^Y_j$ in Eq. (5)).

The assumption of a constant contact rate is questionable under containment measures, such as smart working and remote schooling. Thus, mobility restrictions are taken into account by reducing the contact rate where the restrictions are imposed (see “Modeling non-pharmaceutical interventions”).

### Modeling contact tracing

Similarly, the forces of contact tracing $\mu ^A_i$ and $\mu ^{A'}_i$ are the rates at which asymptomatic subjects of class $A_i$ and $A'_i$ are isolated (Eq. (4i–k)) after contact with subjects that just resulted positive to a Covid-19 test. Considering a 1-day delay, we keep the contact tracing rates constant during the day, at values based on the encounters with subjects that got isolated during the previous day. In formulas, denoting by $\mu ^X_{i,d}$ the contact tracing rate for class $X\in \{A,A'\}$ in group *i* during day *d*, i.e., time $t\in [d-1,d]$, we set

6 $$\begin{aligned} \mu ^X_{i,d}\! = \sum _{j=1}^2 C_{ij}^X a_j \frac{ \int _{d-2}^{d-1} \sum _{k=1}^2 \big (C_{kj}^I \eta I_k+C_{kj}^A \mu ^{A}_{k,d-1} A_k +C_{kj}^{A'} \mu ^{A'}_{k,d-1} A'_k\big ) dt}{ \int _{d-2}^{d-1} \sum _{Y\in {\mathcal {M}}} \sum _{k=1}^2 C_{kj}^Y Y_k\,dt}, \end{aligned}$$

for $d\!\ge \!2$ and $\mu ^X_{i,1}\!\!=\!0$ for the first day of the model simulation. The integral at numerator gives the average presence in the nations of group *j* during day $d\!-\!1$ of people that got isolated by the end of the day (the flow $\eta I_k$ includes the flows entering classes $I^Q_k$ and $H_k$, while both flows $\mu ^{A}_{k,d-1} A_k$ and $\mu ^{A'}_{k,d-1} A'_k$ enter class $A^Q_k$). The integral at denominator is the average number of mobile people present in group *j* in the same day. Their ratio qualitatively measures the chance that an encounter during day $d\!-\!1$ in a nation of group *j* occurs with a subject who got isolated by the end of the day. The contact tracing parameter $a_j$ includes the daily contact rate in group *j* (the same included in the transmission rates $\beta ^Y_j$ and reduced by mobility restrictions) scaled by the probability that a recent contact with a just-confirmed infected is successfully traced back.

### Modeling non-pharmaceutical interventions

Containment measures, including mobility restrictions, are modeled into five discrete levels based on a model reconstruction of the stringency index (SI), a continuous composite indicator of nine metrics proposed by the Oxford Coronavirus Government Response Tracker project^34^. The SI is calculated daily for each nation as a weighted mean score of the metrics, each taking a value between 0 (no restrictions) and 100 (full lockdown).

Because national governments principally decide the containment measures based on hospital occupancy, we reconstruct the average SI of the nations in group *i* based on the fraction $H_i/N_i$ of the hospitalized people. We fitted an autoregressive-exogenous (ARX) linear model using available data, as detailed in SI Sect. S2.9. The ARX model is then used to simulate the SI$_i$ in group *i* from the input $H_i/N_i$. The model output is truncated in the feasible interval [0, 100] and thresholds are equally spaced to define 5 stringency levels ($\text {SL}_i$) of the imposed measures, from the mildest $\text {SL}_i\!=\!1$ to the strictest level $\text {SL}_i\!=\!5$ (level $\text {SL}_i$: $20(\text {SL}_i-1)\!<\,$SI$_i\!\le \!20\,\text {SL}_i$; $\text {SL}_i=1,\ldots , 5$), with $\text {SL}_i=0$ corresponding to no measure imposed. The contact rate and the infection transmissibility in the group are progressively reduced with the stringency level, starting from the values $\beta ^Y_0$ and $a_0$ used in the absence of containment measures and reducing them by one-third at each successive level (i.e., $\beta ^Y_i\!=\beta ^Y_0*(2/3)^{\text {SL}_i}$ and $a_i\!=a_0*(2/3)^{\text {SL}_i}$ in group $i=1,2$). The idea behind is that the mildest measures, like the use of masks in closed spaces, hand hygienization, and social distancing, induce the largest reduction of the infection risk, whereas more severe measures, like mobility restrictions, smart working, remote schooling, up to a strict lockdown, progressively produce smaller reductions.

### Initial and terminal conditions

We simulate model (4) daily for 1 year without vaccinations, followed by 5 years of vaccination campaign. At the end of each day, we update the forces of contact tracing $\mu ^A_i$ and $\mu ^{A'}_i$ in the two macro-groups of nations, $i=1,2$, we check the stringency levels in both groups, and we accordingly update the contact tracing parameters $a_i$ and the transmission rates $\beta _i^Y$ for the next day. We use a stop criterion for the pandemic on the number of confirmed infected worldwide ($I^Q_1\!+\!A^Q_1\!+\!H_1\!+\!I^Q_2\!+\!A^Q_2\!+\!H_2$), with a strict threshold of $10^{-10}$, i.e., less than 1 confirmed infected in our ﻿world. We start ($t=0$, beginning of day 1) with one person exposed per million citizens in both macro-groups, all others being susceptibles ($E_i(0)=10^{-6}N_i$, $S_i(0)=(1-10^{-6})N_i$, $i=1,2$).

## Results

We investigate through simulations how different vaccination campaigns would have affected the evolution of the Covid-19 pandemic. As introduced in “Modeling protection and vaccination”, we characterize the vaccination campaign based on the *access to vaccines* (2) (the global vaccination rate—the fraction of the world population vaccinated daily) and the *inequity in access* (3) (the log of the ratio of the vaccination rates in HI and MLI countries). We investigate under which conditions the pandemic stops within 5 years of vaccination. Finally, we test the robustness of our results to perturbations of several model parameters, with respect to the values obtained in our calibration (see SI Sect.  S2 for the calibration procedures).

### The status quo

**Figure 2:**
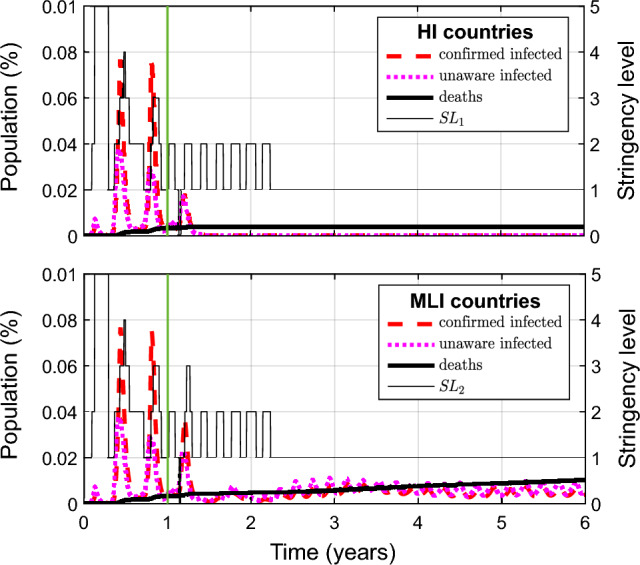
Simulation of the status quo of the Covid-19 pandemic, showing the confirmed infected ($I^Q+A^Q+H$), unaware infected ($A+A'$), and deaths (*D*) on the left vertical axis (population fraction within the group of HI and MLI countries) and the stringency level on the right vertical axis. The green vertical line after 1 year indicates the beginning of the vaccination campaign (vaccination rates: $\rho _1=0.005$ and $\rho _2=0.002$).

We simulate the current scenario, status quo, characterized by vaccination rates $\rho _1=0.005$ and $\rho _2=0.002$, corresponding to access to vaccines 0.0025 (worldwide vaccination rate at 0.25%) and inequity in access $\log _{10}(\rho _1/\rho _2)=\log _{10} 2.5 = 0.3979$. Figure 2 shows the evolution of the main pandemic indicators: confirmed infected ($I^Q+A^Q+H$), unaware infected ($A+A'$), deaths (*D*) and stringency levels. The evolution of all state variables is reported in SI Fig. S8).

The main result is that the pandemic does not stop. The time horizon of 5 years of vaccinations is long enough to appreciate substantial differences between the two macro nodes of nations: in the HI node, the pandemic is kept under control even if it is fueled by infectious mobile subjects from the LMI node, where the pandemic stabilizes in an endemic regime with moderate waves. Note that small infection waves are also present in rich countries in the endemic regime, though not visible at the scale of Fig. 2. Essentially, proceeding with the current vaccination rates, our model foresees herd immunity in rich countries (the class of resistant individuals overcoming the $70\%$ of the population, see SI Fig. S8), but, because of the virus circulation in MLI nations, we are not be able to stop the pandemic.

Looking at the time evolution of the epidemic in the two macro groups, we see that the infection realistically proceeds in waves, even though a quantitative comparison of the timing and intensity between real and simulated outbreaks is not possible without taking seasonality and virus variants into account. However, the number of predicted outbreaks remarkably corresponds to the real evolution of the epidemic in most HI and MLI nations, so far. Three main peaks are indeed observable in aggregate data for HI countries prior to the vaccination campaign: the first of February–September 2020 reached 10 million people (confirmed cases) and caused 0.4 million deaths, whereas 11.5 M cases and 0.13 M deaths are the numbers in our simulation (first peak in Fig. 2, top panel); the second, from October 2020 to February 2021, reached 61 M people and caused 1.3 M deaths, with 281 M cases and 2 M deaths in our simulation (second peak); the third wave is that of March-June 2021, that reached 80 M people and caused 1.6 M deaths, with 243 M cases and 1.8 M deaths in our simulation (third peak). With the start of the vaccinations, our simulation reproduces the fourth wave of August–October 2021. After that, the epidemics remains at very low levels in HI countries. Overall, the total number of confirmed cases and deaths to date (November 25, 2022) is in good agreement with data from HI nations: about 600 M cases and 4 M deaths in our simulation, against 400 M cases and 2.7 M deaths in official data.

### Hypothetical vaccination campaigns

**Figure 3:**
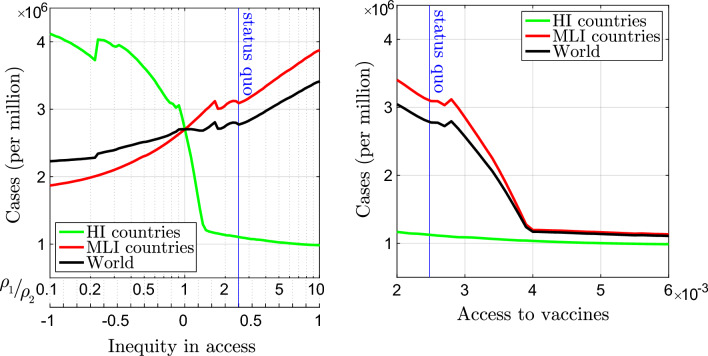
Total Covid-19 cases during the simulation of different vaccination campaigns. In the left panel, the vaccination rates $\rho _1$ and $\rho _2$ in HI and MLI countries are varied to change the inequity in access to vaccines (see Eq. (3); status quo value at $\rho _1/\rho _2=2.5$ indicated by the blue vertical line), while keeping constant at the status quo the global access to vaccines (constant worldwide vaccination rate at 0.25%, see Eq. (2)). The pandemic does not stop in any of these cases. In the right panel, the global access to vaccines is varied (mainly above the status quo value indicated by the blue vertical line), with inequity of access kept constant at the status quo value. The pandemic stops for access to vaccines larger than about 0.004 (worldwide vaccination rate at 0.4%).

We now use the vaccination rates $\rho _1$ and $\rho _2$ as control parameters and we investigate the efficacy of different vaccination campaigns. The consequence of varying the inequity in access (3) is shown in the left panel of Fig. 3, whereas the access to vaccines (2) is fixed to the status quo. If we look at the Covid-19 cases from the points of view of the HI and MLI countries, the optimal strategy is to keep all vaccine doses for themselves. Instead, from a global viewpoint (black line), interestingly, the optimal strategy is to give all vaccines to the MLI countries, so that all the effort should be used to contain the spread of the epidemic in the most populated macro-area. Notice, however, that a sharp transition in the HI cases is present while moving from the status quo toward an equitable vaccine distribution: this is because the current access to vaccines is sufficient to grant herd immunity for HI countries (the less populated macro-area), while the GEA campaign is not. None of the campaign in Fig. 3 left are able to stop the pandemic.

In the right panel of Fig. 3, the effect of varying the access to vaccines (2) is presented, with the inequity in access as in the status quo. Obviously, the higher the global vaccination rate, the better the result. Note, however, the sharp slope change in the MLI and world curves presents at access to vaccines around 0.004. This is the access threshold granting global herd immunity, with the current inequity in access. For larger access to vaccines, the pandemic stops before 5 years of vaccination, with marginal improvements in terms of total cases.

**Figure 4:**
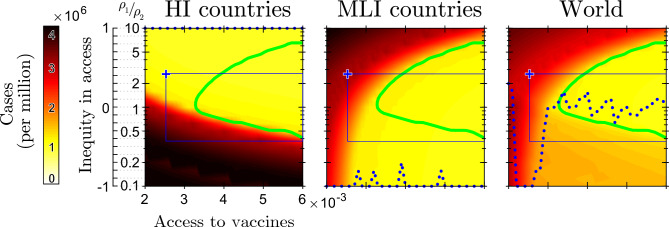
Analysis of the total Covid-19 cases in HI (left), MLI (middle), and worldwide (right) during 5-year vaccination campaigns characterized by different access to vaccines (2) and inequity in access (3). The blue marker indicates the status quo, and is the top-left vertex of the rectangles that includes all the eGEA vaccination strategies. The dotted blue line shows the inequity that minimizes the cases (in HI, MLI countries and worldwide) for any given access to vaccines. Vaccination strategies able to stop the pandemic before 5 years of vaccination are enclosed by the green line.

We finally analyze the effect of all possible vaccination strategies. In particular, we simulate our model for different combinations of access to vaccines and inequity in access between HI and MLI countries. The result in terms of total cases (per million citizens in the single macro-group and at global scale) is shown in Fig. 4 (other pandemic metrics, i.e., deaths, administered vaccines, average stringency index, and pandemic duration are reported in SI Fig. S9). We indicate with a blue rectangle all the eGEA strategies, i.e., those improved in both access to vaccines and equity of distribution with respect to the status quo, that corresponds to the top-left vertex of the rectangle. Those strategies that are able to stop the pandemic in less than 5 years of vaccination are enclosed in the green line.

Figure 4 shows that the pandemic can be brought to an end, provided the intensity of vaccination is large enough. This is per-se a rather trivial result. However, interestingly, the figure shows that the access to vaccines required to stop the pandemic gets minimized by a GEA strategy (zero inequity in access at the left-most point of the green line).

The optimal nature of the GEA strategies can also be seen in terms of minimizing the total cases worldwide (right panel). If the access to vaccines is large enough (essentially enough to stop the pandemic, though this is not necessary; in the analysis of the vaccine efficacy performed in SI Sect. S4, we find GEA optimality even if the access to vaccines is not able to stop the pandemic) GEA strategies are those that minimize the total cases worldwide (see the dotted blue line, which shows the inequity in access that minimizes the metric in the panel). Otherwise, if the access to vaccines is very scarce (less than 0.0022 in Fig. 4, right), the optimal strategy is to focus vaccination in HI countries (the group with a smaller population), i.e., the strategy of preserving a small healthy island, instead of dispersing the few vaccines over a too wide population. On the contrary, for intermediate access to vaccines, actually including the status quo, the best strategy is to reserve most of the vaccine doses for MLI countries (the group with a larger population). Indeed, the minimum of cases worldwide occurs for inequity $-1$ (vaccination rate 10 times higher in MLI countries) for access to vaccines between 0.0022 and 0.0026 (see the dotted blue line at the bottom of the panel) and remains unbalanced toward MLI countries up to access 0.003.

Looking at the total cases in a single macro-group of countries (left and middle panels of Fig. 4), the result is not surprising. The higher the inequity of the vaccination rate in favor of a group, the smaller the number of cases in that group. Actually, in the region with least cases (lightest color; top for HI countries at inequity 1, $\rho _1/\rho _2= 10$; bottom for MLI countries at inequity $-1$, $\rho _2/\rho _1=10$), the number of vaccines is essentially sufficient to quickly cover the population in the group, so that the number of reported cases is roughly the number of cases in the first year of simulation before the start of the vaccination campaign. However, if only one group is protected by vaccination, the pandemic does not stop because fueled by the other group. It stops roughly in the intersection of the lightest regions of the two panels.

### Comparison and robustness

To better understand the benefits and drawbacks of enhancing equity and access to vaccines, we compare the status quo with a selected eGEA scenario. We intentionally select a vaccination-intensive scenario with a slightly improved equity compared to the status quo. The idea is to test what we could have realistically achieved. We therefore take the vaccination rate at 1% in HI countries (12.8 M people per day, double than the current rate) and at 0.5% in MLI countries (33.6 M people per day; more than double, with respect to the current 0.2% rate). This intensive rates (leading to a global access to vaccines at 0.0058) have been reached and even surpassed during the pandemic^4,5, 28^, so that they could have been realistically maintained for a longer period. As for the equity of vaccine distribution between HI and MLI countries, we consider only a slight improvement from the current inequity at $\rho _1/\rho _2=2.5$ to $\rho _1/\rho _2=2$. Vaccine distribution has been constrained by economic and geo-political factors that could hardly go differently at the beginning of the pandemic. A more significant change in the equity of access to vaccines can be planned for the future (Covid-19 and other possibly similar threats).

To compare the selected eGEA scenario with the status quo, we compute the following epidemiological, economic, and social cost metrics, reported in Table 1 separately for HI and MLI countries and aggregated at the world scale: the length of the pandemic; the total number of infected people (in millions, M); the total number of confirmed cases; the fractions of asymptomatics and hospitalized among the confirmed cases; the number of days per year in which the fraction of hospitalized people exceeds the critical threshold $H^*$ (identified in calibration, see SI Sect. S2.4), above which the virus mortality is increased; the total number of deaths; the total number of administered vaccine doses (in billion, B); and, finally, the average Stringency Index. The last columns show the per-cent metric changes from the status quo to the selected eGEA scenario, computed over the 6-year simulation (1-year with no vaccination followed by up to 5-years of vaccination campaign or less in case of pandemic end). The time evolution of the simulation of the eGEA scenario is reported in SI Fig. S10.

**Table 1:** Pandemic metrics in the status quo and in the selected vaccination-intensive eGEA scenario (scaled to a world population *W* of 8 billion people; in the eGEA scenario, averaged quantities are computed by averaging a zero value after the pandemic end and up to the 6-year-horizon of the simulation).

Metric	Status quo			eGEA scenario			${\Delta _{\%}}$		
	HI	MLI	World	HI	MLI	World	HI (%)	MLI (%)	World (%)
Pandemic length (months)	–	–	72	–	–	24.9	–	–	– 65.4
Total cases (M)	1417	20814	22231	1260.8	7434.6	8695.4	− 12.4	− 64.3	– 60.9
confirmed (M)	686.1	7735	8421.1	623.8	3598.8	4222.6	− 9.1	− 53.4	– 49.9
% asymptomatics	48.33	32.9	35.4	49.4	48.3	48.5	3.2	46.8	37
% hospitalized	3.1	4.03	3.9	3	3.1	3.1	− 3.2	− 23.1	– 20.5
Average critical h occupancy (d/y)	49	272	–	39	49.2	–	− 20.4	− 81.9	–
Deaths (M)	4.9	69.2	74.1	4.4	26	30.4	− 10.2	− 62.4	– 58.9
Vaccines (B)	11.7	24.5	36.2	3.2	13.2	16.4	− 73.5	− 46.1	– 54.5
Average Stringency Index	10.4	20.5	–	7.5	7.8	–	− 27.9	− 61.2	–

The systematic negative signs of the metrics gains show the benefits of enhancing global equity and access to Covid-19 vaccines. Not only it could have brought this pandemic already to an end, but it could have done it with tremendous socio-economic advantages, compared to what we qualitatively expect in 5 years of the current vaccination trends. In terms of cases and deaths, the reduction is terrific only for MLI nations, but recall that is the key to stop the pandemic. In terms of the cost of vaccines, the cost and stress for the national health systems (based on critical hospital occupancy), and the cost of the adopted containment measures (based on the average Stringency Index), the advantages are also directly evident in rich countries. The only metric that slightly increases from the current to the selected eGEA scenario is the fraction of asymptomatic individuals among confirmed cases (though the number of such cases decreases), meaning that contact tracing is more effective in the selected eGEA scenario because of the less stringent containment measure.

To check the robustness of the above comparison, we performed a sensitivity analysis of the main cost metrics, by perturbing the three most uncertain parameters: the probability to develop symptoms $\sigma$, the contact tracing rate $a_0$, and the mobility probability *C*. Table 2 reports the changes of the main metrics gains at world scale corresponding to parameter perturbations up to two orders of magnitude (whenever feasible). As a result, the beneficial negative gains are all confirmed for all parameter perturbations. This confirms the robustness of our results in terms of the improvements brought by the eGEA scenario. It does not however mean that the metrics are insensitive to the parameters. In the SI Sect. S4, we present the sensitivity analysis in absolute terms, separately for HI and MLI countries.

**Table 2:** Sensitivity analysis of the worldwide results with respect to the three most uncertain parameters.

	Symptomatic infected $\sigma$						Contact tracing rate $a_0$									Mobility *C*								
	/100	/10	/5	/2	**1 (%)**	$\times 2$	/100	/10	/5	/2	**1 (%)**	$\times 2$	$\times 5$	$\times 10$	$\times 100$	/100	/10	/5	/2	**1 (%)**	$\times 2$	$\times 5$	$\times 10$	$\times 100$
Length	− 65.6	− 63.7	− 63.1	− 63.2	− 65.4	− 66.1	− 64.6	− 64.7	− 64.7	− 65.0	− 65.4	− 65.4	− 65.8	− 66.3	− 66.1	− 65.3	− 65.3	− 65.3	− 65.3	− 65.4	− 65.3	− 65.3	− 65.3	− 65.4
Cases	− 69.7	− 69.9	− 69.8	− 70.8	− 60.9	− 53.8	− 63.5	− 63.3	− 63.4	− 63.7	− 60.9	− 65.6	− 66.8	− 64.2	− 60.3	− 64.3	− 64.3	− 64.3	− 64.3	− 60.9	− 65.2	− 64.3	− 64.3	− 64.4
Deaths	− 65.8	− 64.9	− 64.2	− 65.1	− 58.9	− 49.6	− 58.1	− 58.0	− 58.1	− 58.3	− 58.9	− 60.2	− 61.3	− 58.3	− 52.4	− 58.9	− 58.9	− 58.9	− 58.9	− 58.9	− 60.2	− 58.9	− 58.9	− 59.3
Vaccines	− 55.0	− 50.2	− 48.9	− 49.2	− 54.5	− 56.5	− 52.4	− 52.7	− 52.9	− 53.6	− 54.5	− 54.8	− 55.8	− 57.0	− 56.6	− 54.5	− 54.5	− 54.5	− 54.5	− 54.5	− 54.5	− 54.5	− 54.5	− 54.7

## Discussion

We proposed a deterministic model for the global spread of the Covid-19 pandemic, including vaccination, isolation of symptomatic subjects, the tracing of potentially infectious contacts, and the containment measures imposed by local governments. Analyzing public data^4,5^, we found no significant differences in the epidemiological parameters between high-income (HI) and mid-to-low-income (MLI) nations, whereas the difference in access to Covid-19 vaccines has been so far quite remarkable (HI countries using nearly 40% of the production while accounting for only 16% of the world population^5^). We therefore aggregated countries into two groups, HI nations in group 1, and all others (MLI) in group 2, connected with a symmetric weak mobility, and we investigated the role of different vaccination strategies on the evolution of the pandemic. In particular, we studied the interplay between the *access to vaccines*, meant as the global effort to produce and administer Covid-19 vaccine worldwide, and the *inequity in access* between HI and MLI countries, relative to their populations. We labeled eGEA (enhanced global equity and access) the vaccination strategies that, compared to the status quo, improve both access and equity in the distribution of Covid-19 vaccines.

The main result is that a considerable access to vaccines is needed to stop the pandemic, and that the required access gets reduced the more equity in access is granted. Indeed, with the current inequity in access, the increase in vaccine access needed to stop the pandemic predicted by the model is about $60\%$, vs. $32\%$ if we adopt the GEA strategy (equal vaccination rates in HI and MLI countries).

The proposed model contains many simplifying assumptions, so only a qualitative match with the data can be expected. For example, the model assumes perfect tests (swabs), which were few and far than perfect, with significant false negatives, in the first year of the pandemic^52,53^. This results in the first three waves being more intense in our simulation than in reported data. Furthermore, the model uses constant parameters, and therefore does not consider the virus variants that partly eluded the vaccine protection, starting with the Omicron variant that appeared at the end of 2021. The large waves observed in 2022 are therefore not reproduced by the model. On the other hand, the model is able to capture the overall behavior of the pandemic, showing that the infection proceeds in waves and capturing the overall numbers (both for cases and deaths) that have been recorded.

In conclusion, the model validation of the status quo is qualitatively good. Despite the mentioned quantitative discrepancies, we think that the model can usefully inform about the entity of the Covid-19 pandemic, especially in low-income countries where official data are not reliable. Besides the compulsory nature of our vaccination mechanism and the full protection against infection assumed for an average period of 6 months per dose (in accordance with data provided by the pharmaceutical companies at the beginning of the vaccination campaign), the model can be used, in retrospective, to investigate the differences, with respect to the status quo, of eGEA vaccination strategies, which is the main goal of this study. 

The model can also be used for discussing future predictions, provided the hypothesized vaccination rates are feasible to implement (taking vaccine hesitancy into account) and vaccines are kept updated and effective on the last variants. Specifically, the model shows that in the status quo the pandemic remains significantly active only in MLI countries, with moderate waves that fuel small waves in HI countries. Rich countries continue the vaccination campaign to renew the waning protections, preventing the epidemic from restarting. Even if the pandemic is technically not over, we can consider the current scenario to endemic.

We first analyzed how a redistribution of the current amount of vaccines would affect the pandemic evolution, in line with previous studies^26,27^. We found that, independently from the equity in access, the current access to vaccines is insufficient to stop the pandemic. The minimization of cases and deaths worldwide would require concentrating the vaccinations on MLI countries, which have the largest populations. Since instead most of the vaccines are distributed in HI nations, herd immunity is achieved there. Giving more vaccines to LMI countries, we would have experienced fewer cases and death globally ($-8\%$ cases globally), but herd immunity would have not been achieved in HI countries ($-18\%$ cases in MLI nations but $+134\%$ in HI nations). This is in agreement with the results presented in^26,27^. Interestingly, the number of deaths in the GEA scenario with the current access to vaccines is fully comparable with the one computed in the analysis by Moore et al.^26^, even if the model and its calibration are different.

We then investigated the interplay of both control parameters, access to vaccines and inequity in access. We found a two-fold optimality of the GEA strategies. As stated in our main result, the minimum access required to stop the pandemic requires equity in access. But even if the access is taken larger, to further limit the impact of the pandemic, the duration is minimized by almost GEA scenarios. Along with the duration, also other indicators of the global impact of the pandemic get minimized, such as total cases and deaths worldwide and the average stringency index, measuring the socio-economic costs of the pandemic. The pattern of the optimal inequity for increasing access is in favor of HI countries (the less populated group) if vaccines are very scarce, in favor of MLI countries (the larger group) for intermediate availability of vaccines, GEA if access to vaccines above a threshold that is almost the one for which the GEA strategy stops the pandemic.

To get an idea of the highest benefits that could have realistically been obtained with an eGEA vaccination strategy, we have compared the status quo with a vaccination-intensive strategy characterized by vaccination rates that have been shown possible (and even exceeded) during 2021–22 in both HI and MLI countries. Compared to the status quo, these rates result in a global $+130$ increase in access to vaccines and in a moderate $20\%$ reduction of inequity (from a 2.5:1 to a 2:1 ratio of the vaccination rates in HI and MLI countries, realistically representing the unavoidable inequity in the economic and political power).

In the proposed eGEA scenario the pandemic stops in about a year, essentially before the appearance of the Omicron variant, thus justifying the assumption of fully protective vaccines. The benefits with respect to the status quo are terrific, especially in terms of cases ($-60.9\%$) and deaths ($-58.9\%$) worldwide. Further global benefits can be gained with more equity in access. However, the benefits at the proposed inequity (with vaccination rate in MLI countries at half of the rate in HI nations) are not far from the GEA optimal values ($4.1\%$ more cases and $4.2\%$ more deaths than the optimal case, see SI Fig. S11 and SI Tab. S4).

The robustness of the benefits gained by adopting our eGEA scenario has been tested by extensive sensitivity analysis with respect to the most uncertain model parameters (see Table 2). We first explored the parameters representing the mobility of people between HI and MLI countries and the intensity of contact tracing, which can be controlled by the governments. The analysis shows that the gains of our selected eGEA scenario compared to the status quo remain essentially unchanged worldwide independently of other lines of intervention (the sensitivity analysis for HI e MLI nations separately is presented in SI Sect. S4).

We then investigated the robustness of our findings concerning vaccine efficacy and the duration of protection. These two factors play a crucial role in assessing the potential of eGEA vaccination strategies and are topics of intense debate within the scientific community^29–33^. Initial declarations from leading pharmaceutical companies placed vaccine efficacy at 90–95%, though this was before the emergence of the Omicron variant^54,55^. Despite continuous updates to vaccines^56^, maintaining such high efficacy against infection is challenging due to the persistent emergence of new variants. Conversely, evidence suggests that the protection against severe outcomes of the disease remains consistently high and stored for an extended period in our immune system^57^. Accordingly, we calibrated hospitalization and death rates over a multi-year horizon, reflecting ongoing exposure to either vaccination or infection. This approach aimed to maintain a relatively constant level of protection against severe outcomes for everyone, irrespective of individual vaccination or exposure history. We systematically varied both vaccine efficacy against infection and the duration of the protection: the detailed results can be found in the SI Sect. S4, with a summary provided below.

As for the efficacy, we consider that not all vaccinated susceptible gain resistance against the virus, but only a fraction *e*, that is a new parameter representing the vaccine efficacy against infection. The remaining fraction $(1-e)$ has the same infection risk of susceptible, but is not eligible for vaccination until the waning of the presumed protection. We find that for waning rate larger than 3 months and vaccine efficacy larger than 80% our results remain valid. The proposed eGEA strategy stops the pandemic with still interesting benefits. With less effective vaccines, the eGEA strategy is not able to stop the pandemic, with a significant decrease of the gains with respect to the status quo. Interestingly, the optimality of GEA vaccination strategies remains confirmed even for lower vaccination efficacy. For efficacy between 66 and $80\%$, vaccination-intensive strategies stop the pandemic, with GEA minimizing the required access to vaccines. But even for lower efficacy, GEA strategies remain optimal, in terms of total cases worldwide, if the access to vaccines is large enough (see the SI Fig. S12) In other word, the pattern of the optimal inequity for increasing access remains qualitatively the same that we found with fully protective vaccines. Finally, consider that our analysis is performed with constant parameters during the pandemic evolution, so that it assumes the reduced vaccine efficacy since from the beginning of the vaccination campaign, whereas, in reality, the drop in efficacy occurred with the emergence of the Omicron variant. The corresponding drop in the benefits brought by eGEA strategies reported by our analysis is therefore overestimated.

The analysis of partially protective vaccines confirmed GEA as a target to be pursued to optimize the global impact of the pandemic. This means that the significance of our model goes beyond retrospective. It shows how global cooperation could improve the management of infectious diseases, in the current Covid-19 epidemic, as well as for future potential pandemics. Second, the benefits of selected eGEA vaccination strategies are still at hand for Covid-19, provided we keep an effort in maintaining vaccines updated and in developing a broadly protective “universal” vaccine (against all betacoronaviruses)^9^. By simulating our model to date in the current scenario and then switching to the proposed eGEA scenario, the model foresees, with fully protective vaccines, the pandemic end in about 12 months from now. All the analyzed socio-economic metrics remain advantageous with respect to the status quo. Clearly, the sooner we change the vaccination strategy, the greater the benefits.

This is an important, model-informed message for our policymakers.

To implement an eGEA scenario, the two main issues are to increase the vaccine production worldwide and to improve the equity in the distribution and administration facilities. Since the vaccination rates we proposed in the selected eGEA scenario have been already shown feasible in both in HI and MLI countries, the principal obstacle remains how to motivate the pharmaceutical companies that hold the IP rights on Covid-19 vaccines. The selected eGEA scenario requires a more than doubled production daily, but the overall numbers on a longer timescale get drastically reduced by the early stop of the pandemic, in comparison to the status quo. Looking at our simulations, the total administrated doses in the eGEA scenario is 16.4 billions worldwide, compared to 36.2 billions used over 5 years in the status quo. Fewer vaccine doses directly mean less profit. We can therefore use our model to quantify the loss for the rights holders in the case of an IP waiver, one of the tools highly discussed to increase vaccine production. If we assume that the production cost of a vaccine dose is 15 USD, the IP cost is 10 USD (thus obtaining the average price of 25 USD/dose^58^), the cost for the 5-year vaccination campaign in the status quo is 292.5 billion USD for HI countries and 612.5 billion USD for MLI ones, with a total profit for the pharmaceutical companies in the 5 years of 362 billion USD. In the selected eGEA scenario under IP waiver (in which only the production cost is paid at no profit for the companies), the cost for vaccines is 48 and 198 billion USD for HI and MLI countries, with a saving of 244.5 and 414.5 billion USD, respectively, with respect to the status quo. The lost profit is therefore covered by the saving on vaccines. Actually, 67.5% of the lost profit is covered with only the saving of HI countries. This justifies and supports compensation mechanisms to negotiate with the IP rights holders the option of an IP waiver, or alternative voluntary/compulsory licensing agreements.

Besides the issues related to production and licensing, there are other important barriers that can limit the implementation of eGEA vaccination strategies, here not taken into consideration for the sake of simplicity. For example, manufacturing constraints^59^, affordability (even if prices should decrease with increasing production), logistical barriers (e.g., issues with vaccine supply chains^60^), healthcare system constraints (e.g., reaching remote villages, number of trained healthcare workers), pre-allotment preparedness^61^, and vaccine hesitancy^62,63^.

Future steps of research will address a more realistic Covid-19 vaccination campaign to estimate the benefits of eGEA vaccination strategies more realistically. For example, middle- and low-income countries could be separated, to better take into consideration the effective health facilities of poor countries, currently overestimated by our model because averaged with those of middle-income countries. Epidemiological parameters and vaccine protection could be varied in time, to track the emergence of new virus variant and vaccine updates. Vaccine protection could also be made age-dependent^26^ and discriminate between effectiveness against infection and severe outcomes^64–67^. Vaccine hesitancy could be considered by adding a class of subjects who do not want to get vaccinated and by modeling the related socio-cultural dynamics^62,63^. Finally, from the viewpoint of the control policy, also the vaccination rates could be adjusted in time to optimally govern the pandemic^27^ in accordance with prescribed targets. These new approaches address the issue that controlling outbreaks by herd immunity may be an elusive goal with Covid-19, because protections could last briefly (especially in older people), or if escape mutants emerge. Infections may continue indefinitely, albeit hopefully at a low endemic level^68^. We expect that the general conclusion will be the same drawn here. To stop the Covid-19 pandemic, we need an efficient vaccine^9,56^ and an intensive GEA vaccination campaign that eradicates the virus before an escape mutant undermines again vaccine efficacy.

### Supplementary Information

Supplementary Information.

## Data Availability

The datasets analysed during the current study are available in the Our World in Data repository, https://ourworldindata.org/coronavirus.
