# Supplementary material for: Model-informed health and socio-economic benefits of enhancing global equity and access to Covid-19 vaccines
Source: Sci Rep. 2023 Dec 7;13:21707. doi: 10.1038/s41598-023-48465-y (PMC10709334; doi:10.1038/s41598-023-48465-y)
Supplement: Supplementary file 1 — Supplementary Information. [file 41598_2023_48465_MOESM1_ESM.pdf]

Supplementary Information

Model-informed health and socio-economic benefits of enhancing Global Equity and Access to Covid-19 vaccines

Matteo Italia, Fabio Della Rossa, and Fabio Dercole

Department of Electronic, Information, and Bioengineering,
Politecnico di Milano, Milano, Italy.

Corresponding author: matteo.italia@polimi.it

S1 Model resuming tables

Tab. S1 resumes the model variables, one for each compartment, and Tab. S2 the meaning and value of the model parameters.

S2 Calibration procedures

To calibrate the model parameters, we partly rely on the estimates presented in Gatto et al. [21] for the early stage of the pandemic in Italy as a representative HI country (see also the refs. therein). For the parameters that significantly changed with respect to the early stage, i.e., those linked to our ability to cure and detect the virus, and for those related to vaccination, we perform new calibrations (data source: ourworldindata.org [4, 5] if not otherwise specified). All model parameters and their values are summarized in Tab. S2.

S2.1 Epidemiological parameters in HI and MLI countries

We investigate if the Covid-19 evolution is similar in high-, middle-, and low-income countries, or if different epidemiological parameters should be adopted among the different groups of nations. Since we extend the model proposed by Gatto et al. [21] for the initial spread of Covid-19 in Italy (a rich country), we first test the hypothesis that the epidemiological parameters for middle and low-income countries are similar to those used for rich nations. To this end, we compare the strength and temporality of the Covid-19 outbreaks in the three groups of high-, middle-, and low-income countries. For each country (place of the outbreak), an outbreak is identified by a peak of the number of weekly new cases per million citizens. More precisely, the strength of the outbreak is a daily local maximum of the new confirmed cases of the last seven days (only considering maxima above the epidemiological threshold of 150 cases per million per week [57, 58]) that is

Variable	Definition
S_i, S_i'	susceptible (non infected, unprotected/partly protected)
E_i, E_i'	exposed (infected during latency, non infectious)
P_i, P_i'	presymptomatic (infectious, prior to possible symptoms onset)
I_i	unconfirmed symptomatic (infectious)
I_i^Q	quarantined symptomatic (infectious, isolated)
A_i, A_i'	untraced asymptomatic (infectious, not developing symptoms)
A_i^Q	quarantined asymptomatic (infectious, not developing symptoms, isolated)
H_i	hospitalized (infectious, isolated)
R_i, R_i'	unaware/aware resistant (non infected, fully protected)
D_i	dead

Table S1: State variables of the model. All variables are expressed as fractions of the world population. The subscript i denotes the country of residence ($i = 1$ for HI countries; $i = 2$ for MLI ones). The prime-superscript denotes the classes of individuals who recently received a dose of vaccine and are developing resistance (except for the class R', that includes individuals aware of being resistant by vaccination or recovery from the disease). Classes I^Q, A^Q, and H include all subjects that are currently confirmed to be infected; they are all assumed to be isolated.

Par.	Value	Name (Unit)	Description
N_1<br>N_2	$0.16W$<br>$0.84W$	Population size in group i	Fractions of the world population W resident in HI and MLI countries (groups 1 and 2)
δ_E<br>δ_P	1/3.32<br>1/0.75	Progress rate from class $X \in \{E, P\}$ (day^{-1})	$1/\delta_X$ is the average time spent in class X [21]
γ_I<br>γ_A<br>γ_H	0.07<br>0.14<br>0.07	Recovery rate in class $X \in \{I^Q, A, A', A^Q, H\}$ (day^{-1})	$1/\gamma_X$ is the average duration of the symptomatic/asymptomatic/hospitalized phase before recovery [21]
σ	0.25	Fraction of symptomatic infected	Probability of developing symptoms [21]
β_0^P<br>β_0^I<br>β_0^A	3.85<br>0.131<br>0.127	Transmission rate in class $X \in \{P, I, A\}$ (day^{-1})	Contact rate multiplied by the probability that the infection is transmitted during a contact with a subject in class $X \in \{P, I, A\}$
η	1	Isolation rate of symptomatic infected (day^{-1})	$1/\eta$ is the average time from symptom onset to infection certification
ζ	0.94	Fraction of home-quarantined symptomatics	Probability of not needing hospital care
α	0.017	Mortality rate	Applied only to class H. Increased by 35% for the fraction of H_i exceeding $N_i\, H^* \times 10^{-6}$
H^*	130	Hospital capacity threshold (subjects per million people)	Threshold taking into account hospital capacity saturation in HI nations
a_0	38.5	contact tracing rate	Contact rate multiplied by the probability of contact tracing [56]
ρ_1<br>ρ_2	0.005 | 0.01<br>0.002 | 0.005	Vaccination rates in the status quo | eGEA scenario (day^{-1})	$\rho_i N_i$ is the total number of vaccine doses administered per day in the nations of group i [5]
ν	1/14	Protection developing rate (day^{-1})	$1/\nu$ is the average time to develop full protection after receiving a vaccine dose [36]
ω	1/180	Protection waning rate (day^{-1})	$1/\omega$ is the average duration of the protection once in the resistant classes R and R' [37, 38]
C_{ij}^X	0.0002	Mobility probability	Probability for an individual resident in group i in class X to be present in group $j \neq i$

Table S2: Summary of the model parameters and their calibration.

not exceeded in the previous and posterior 45 days. The temporality of the outbreak is the time (in days) from the previous peak (from the first case in the country for the first peak).

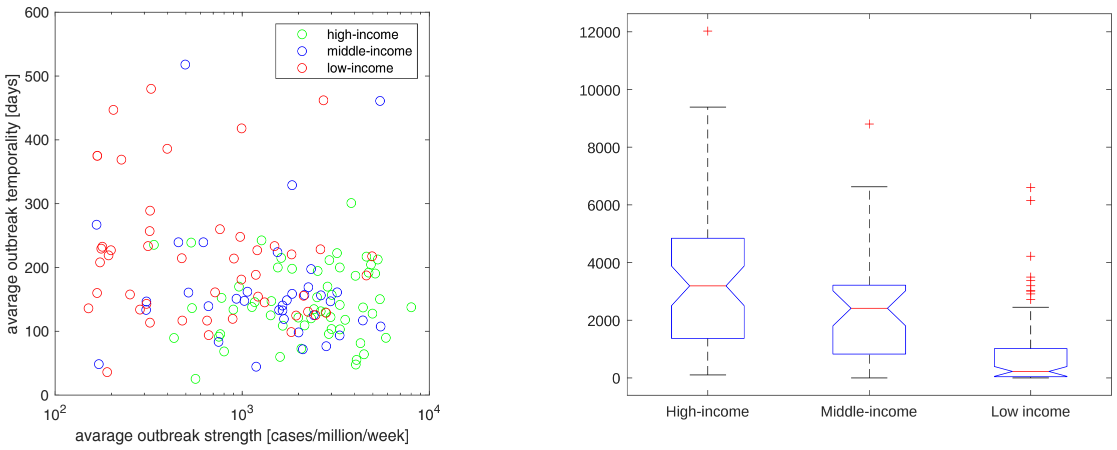

(a) (b)

Figure S1: Covid-19 outbreaks in high-, middle-, and low-income countries. Panel (a) reports the average outbreak strength (measured in cases/milion/week, in log scale) versus the average outbreak temporality (measured as days from the previous peaks); green, blue, and red dots represent low-, middle-, and high-income outbreaks, respectively. Panel (b) reports the box plot of the average outbreak strength for the three groups.

Fig. S1(a) shows the average outbreak for each nation (up to September 2021, to avoid the virus variants for which the hypothesis of a full vaccine protection is questionable). A visual inspection highlights that data include outbreaks of moderate intensity for most low-income countries (red dots), whereas outbreaks are similar in middle- and high-income countries, as further highlighted by the box-plots reported in Fig. S1(b). This is confirmed by statistical tests. A one-way ANOVA [59] (excluding outliers), confirms that there is a statistical difference between the outbreak average strength in the three groups (p-value of the no-difference hypothesis: $< 10^{-12}$, p-value of the Kolmogorov-Smirnov test on residual normality: 0.09, p-value of the Box test for residual homoscedasticity: 0.06). The multiple comparison marginal analysis [60] confirms that low-income countries have a significantly lower average with respect to the other two groups (95%-CI for the average of the three groups: low-income, $[-221, 512]$ note the even negative lower bound; middle-income, $[1797, 2996]$; high-income, $[2906, 3596]$). In the absence of relevant reasons to justify such a difference, and also excluding climatic factors (the same statistical difference is observed among countries at similar latitudes), we conclude that Covid-19 infection certification is not reliable (largely underestimated) in low-income countries.

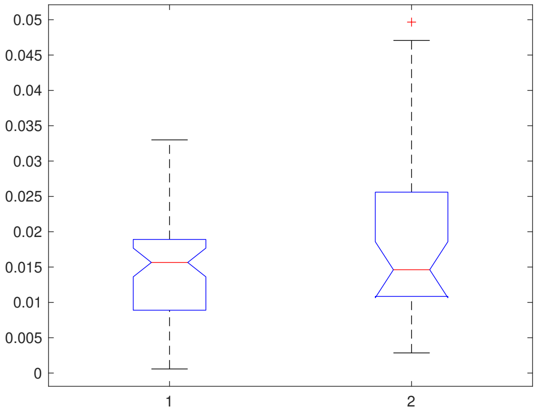

Figure S2: Boxplots of the ratios between total deaths and cases among high-income (group 1 on the left) and middle-income (group 2) nations.

To further test the data similarity between high- and middle-income countries, we run the multi-variate MANOVA test [61] jointly on the strength and temporality of the outbreaks (p-value of the no-difference hypothesis: 0.12; p-values of the Kolmogorov-Smirnov test on data normality in each group: 0.65 and 0.24; p-values of the Box tests for homoscedasticity between the aggregated data (both groups) and the single groups: 0.56 for high-income countries; 0.33 for middle-income countries). We therefore conclude that there is no evidence for differences in the spread and evolution of Covid-19 in high- and middle-income countries, so that, discarding the unreliable data from low-income ones, we use the same epidemiological parameterization in the two macro groups.

Finally, because data on outbreaks do not explicitly account for mortality, we also compare the ratio of Covid-19 total deaths over the total number of confirmed cases across high- and middle-income countries (mortality data from low-income countries are not considered because unreliable, as well as data on contagions). The box-plots of the data, reported in Figure S2, suggest that no significant difference is present between the two groups. This is confirmed by an ANOVA test (p-value of the no-difference hypothesis: 0.17; p-values of the Kolmogorov-Smirnov test on data normality in each group: 0.32 and 0.24; p-values of the Box tests for homoscedasticity between the aggregated data (both groups) and the single groups: 0.14 for high-income countries; 0.10 for middle-income countries). These analyses allow us to conclude that neither mortality data show statistical evidences of difference in the two groups.

S2.2 Basic reproduction number R_0, progress rates δ_E, δ_P, recovery rates γ_I, γ_A, γ_H, and fraction of infected subjects developing symptoms σ

We take from Gatto and coauthors [21] their estimates of the basic reproduction number $R_0 = 3.6$, of the progress rates δ_E and δ_P, of the recovery rates γ_I, γ_A, and γ_H, and of the fraction σ of infected subjects developing symptoms (see Table S2). In particular, the latter parameter is rather uncertain, due to the untraceable nature of asymptomatic infections. Recently, a meta-analysis of several datasets distinguishing symptoms level among confirmed cases revealed a 40% fraction of totally asymptomatic subjects [62]. The estimated fraction however misses the untraced subjects, so the asymptomatic fraction over all infections is expected to be larger (the value used by Gatto and coauthors is $1-\sigma = 0.75$). Given the intrinsic uncertainty, we include parameter σ in the list of those for which a sensitivity analysis of our results is performed.

From R_0, we derive the transmission rates β_0^P, β_0^A, and β_0^I in the absence of stringency measures. Specifically, we assume the same ratios between the transmission rates used by Gatto and coauthors and we use the same model formula for computing β_0^P from a given R_0 (see [21] Supporting Information), as it is computed at the disease-free equilibrium, where the models are equivalent. The resulting transmission rates are however slightly different because of different values of two other parameters: the isolation rate η (at home or in hospital) of symptomatic subjects and their mortality prior to isolation. The first has been increased, due to changes in people awareness and behavior from the early stage of the pandemic (the average time $1/\eta$ from symptom onset to isolation has been reduced from 4 to 1 day), while the second has been set to zero, neglecting those deaths (infected subjects can only die from class H).

S2.3 Fraction of hospitalized symptomatics $1 - \zeta$

To estimate the fraction of home treated (quarantined) symptomatic subjects ζ, we actually look at the complementary fraction $1 - \zeta$ of hospitalized symptomatics. We use the weekly hospital admissions of Covid-19 patients and the weekly new Covid-19 cases, the latter corresponding in the model to the weekly inflow to the classes A^Q, I^Q, and H. However, parameter ζ only discriminates symptomatics in the model, so that, to remove asymptomatic cases, we use the estimate of 60% incidence of asymptomatic infections among the tested population reported in [62]. Denoting the weekly inflow to a class with the 'in'-subscript, we can therefore link data and model by writing $I^Q_{\text{in}} + H_{\text{in}} = 0.4$ (new weekly cases) and hence

$$1 - \zeta = \frac{H_{\text{in}}}{I^Q_{\text{in}} + H_{\text{in}}} = \frac{\text{(new weekly hospitalized cases)}}{0.4\text{(new weekly cases)}}.$$

In conclusion, we estimate $0.4(1-\zeta)$ as the least square linear regressor (with zero intercept) between the new weekly hospitalized cases and the new weekly cases. We used data from 21 high-income nations, for which both data series are available. The resulting regression is shown in Figure S3, yielding to the estimate $\zeta = 0.94$. The determination coefficient $R^2 = 0.23$ is not so high, essentially because the fraction of hospitalized

cases varied significantly between the first and successive infection waves and dropped substantially with the recent less severe virus variants (see dots below the regression line in the figure). Our model, however makes use of constant parameters, so that we keep the average fraction along the pandemic.

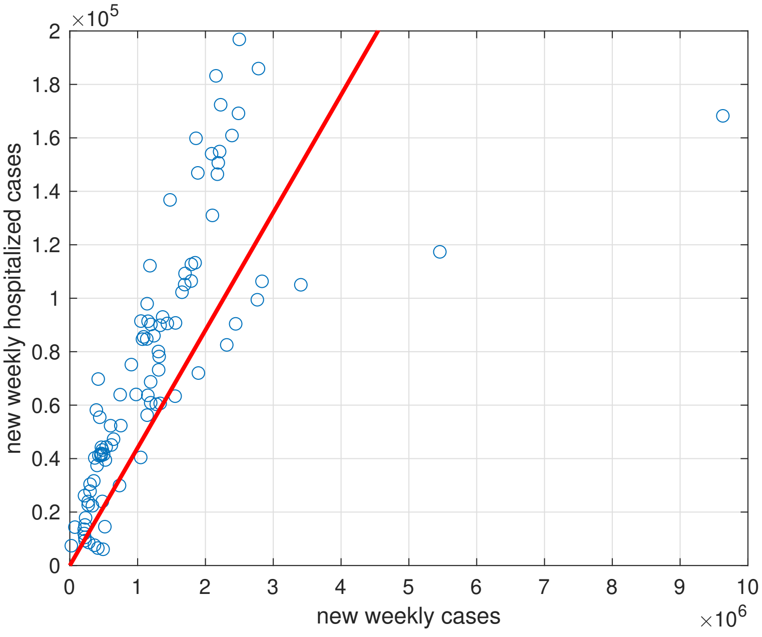

Figure S3: Regression between the weekly new hospitalization and the weekly new cases. Data aggregated, from March 5, 2020, up to February 3, 2022, for the following 21 high-income nations (ISO code): BEL, CHL, CZE, DNK, EST, FRA, DEU, IRL, ISR, ITA, LVA, LUX, MLT, NLD, NOR, SVN, KOR, ESP, CHE, GBR, and USA. In red, the linear regression we used to estimate parameter ζ.

S2.4 Mortality rate α

In our model, infected subjects die only from the hospitalized class H (see eqs. (4)l-m). We therefore estimate the mortality rate α by looking at the ratio between daily deaths and hospital occupancy in the same day. Data are available daily for 34 high-income nations. We apply a 7-day moving average and aggregate data over the considered countries to avoid weekly oscillations and attenuate other noise sources. The least square linear regression (with zero intercept) between these two series gives a slope of 0.019 ($R^2 = 0.82$).

However, data show a super-linear behavior, representing the fact that the mortality rate is higher when hospital capacity gets saturated (Fig.S4). We have therefore fitted a piecewise linear model, with a slope α applied to hospitalized people up to a capacity threshold H^* (expressed in hospital beds per million people) and a higher slope α^* applied over the threshold. The obtained least square estimates are $\alpha = 0.017$ and $\alpha^* = 0.023$ (a 35% mortality increase), with an occupancy threshold of about $H^* = 130$ patients per million

citizens. The estimates have been obtained by varying the threshold H^* with steps 10 beds (per million people) and computing the linear regressions separately for data below and above threshold. The piece-linear fitting is shown in Fig.S4. The Fisher test on the variance of the squared residuals refuses the null hypothesis that the linear model explains the data better than the piece-wise one (p-value $< 10^{-12}$).

The optimal threshold H^* is indeed of the order of the hospital occupancy recorded in the first wave of infection and considered critical for the national health systems in rich countries. It essentially corresponds to the average intensive care beds capacity in high- and middle-income nations. Recalling that the state variables of our model represent actual populations in the two macro-groups of high- (HI) and mod-to-low-income (MLI) nations, the threshold for the variable H_1 to be used in eqs. (4)l-m is $N_1 H^* \times 10^{-6}$. In absence of reliable data for low-income countries, we use the same threshold H^* in the second group, so that the threshold for H_2 is $N_2 H^* \times 10^{-6}$, where recall that $N_1 = 0.16\,W$, $N_2 = 0.84\,W$, W being the world population.

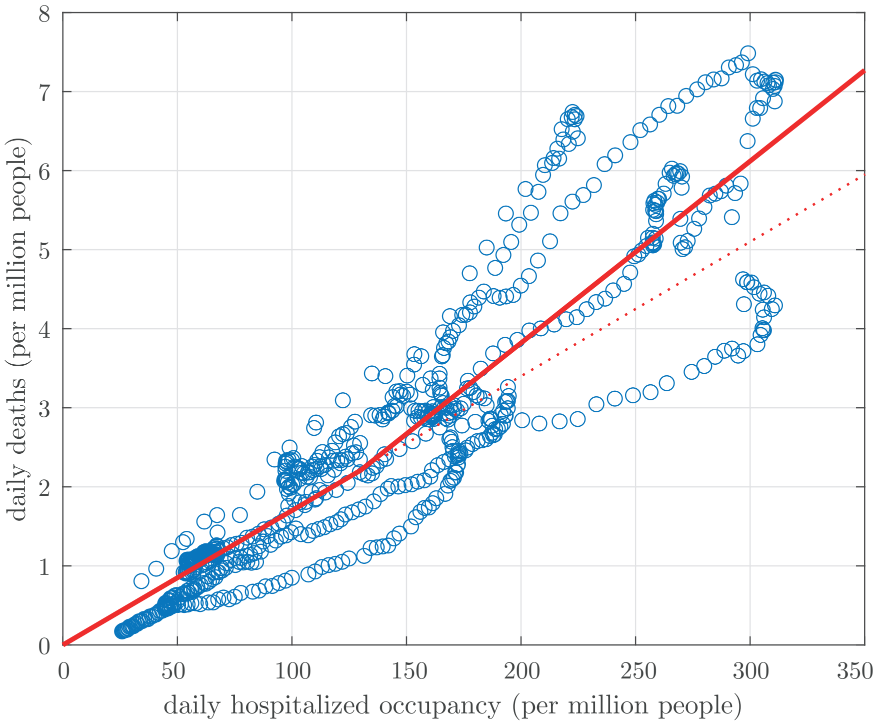

Figure S4: Piecewise-linear regression between (7-day-averaged) daily deaths and hospital occupancy. Data aggregated, from March 5, 2020, up to February 3, 2022, for the following 34 high-income nations (ISO code): AUS, AUT, BEL, CAN, HRV, CYP, CZE, DNK, EST, FIN, FRA, HUN, ISL, IRL, ISR, ITA, JPN, LVA, LTU, LUX, MYS, MLT, NLD, NOR, POL, PRT, SGP, SVK, SVN, ESP, SWE, CHE, GBR, and USA.

S2.5 Contact tracing rate a_0

As for the contact tracing rate a_0 at zero stringency measures, we qualitatively identify the ratio β_1^P/a_1 for the HI group on a small (but representative) dataset at the first level of stringency. This quantity is liked to a_0 by our modeling of the containment measures (see Sect.2). It indeed results $\beta_1^P/a_1 = \beta_0^P/a_0$, independently of the imposed restrictions in group 1 (because the SL_1-dependent factors in β_1^P and a_1 cancel out), from which we get $a_0 = \beta_0^P/(\beta_1^P/a_1)$. Recall that β_1^P/a_1 is the ratio between two probabilities (because the daily contact rate included in both β_0^P and a_0 cancels out in the ratio): the probability of getting infected given a contact with a presymptomatic subject, and the one to be traced given a contact with a subject who is confirmed infected by the end of the day. It can be estimated as the average ratio between the number of secondary cases generated by a confirmed infection and the number of people that have been tested as a consequence of the primary case (e.g., if all contacts are traced, then the probability at the denominator is 1 and the ratio just gives the probability to get infected given the contact; otherwise it gives a larger value). As a representative case, we found a local dataset on schools surveillance made public by Regione Veneto in Italy [56] (to the best of our knowledge this is the only data sample that allows the distinction of primary from secondary cases). Data report 4832 primary cases that caused 83660 tested people (students and school personnel) and 2793 secondary cases, in a stringency scenario imposing the use of masks and distancing in closed spaces. The resulting ratio is almost 30. The considered scenario is however one of the most severely traced in rich societies, so that we qualitatively set $\beta_1^P/a_1 = 1/10$, resulting in $a_0 = 38.5$. Because of the extreme locality of the dataset and given the uncertain role attributed to contact tracing on the long-term evolution of the epidemic [63, 64, 65, 66], we include parameter a_0 in the list of those for which a sensitivity analysis is performed.

S2.6 Vaccination rates ρ_1 and ρ_2

Looking at Covid-19 vaccine administration, there is a significant difference between HI and MLI countries. In the status quo, i.e., the current scenario, vaccine production is the limiting factor, allowing the administration of a vaccine dose to about 0.5% and 0.2% of the population per day in HI and LMI countries, respectively, so that we set $\rho_1 = 0.005$ and $\rho_2 = 0.002$.

Indeed, the available vaccination data show a clear difference between HI and MLI nations. In HI countries, the vaccination campaign proceeded with an average rate (over 2021) of 0.46 % of the population injected a dose per day (standard dev. 0.18). We therefore set the rollout parameter ρ_1 to 0.005 in the status quo.

For MLI nations, we exclude China, because China decided to produce its own vaccine, calling itself out from the competition to acquire the vaccines on the private market. In the current scenario, China looks like an outlier, with very high vaccination rates in mid-2021 (see Fig. S5). Considering vaccination data

from China would therefore raise the average vaccination rate in MLI nations, unrealistically showing the current inequity in access more globally equitable than it has been so far. Aggregating vaccination data in MLI nations (without China), see green line in Fig. S5, results in an average vaccination rate (over 2021) of 0.24 % of the aggregated population (standard dev. 0.018). We therefore set the rollout parameter ρ_2 to 0.002 in the status quo.

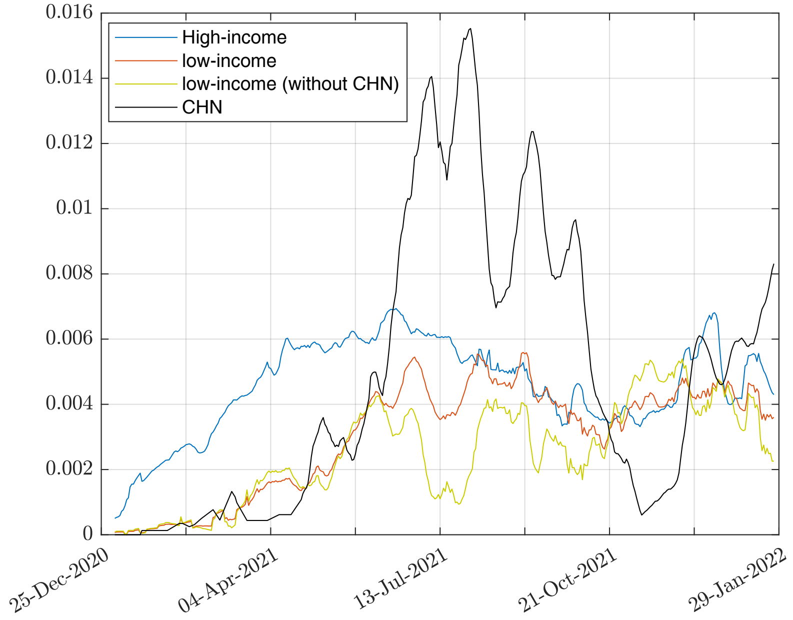

Figure S5: Time series of the vaccination rate (fraction of the population vaccinated per day) in the two groups of HI and MLI countries, along 2021. Data from China are separately plotted to show how China results as an outliers.

Note that the rollout parameters ρ_1 and ρ_2 are the control variables. Indeed, it represents the adopted vaccination campaign, which is decided and controlled by central governments. The rollout parameter defines both the access to vaccines ($(N_1\rho_1 + N_2\rho_2)/W = 0.16\rho_1 + 0.84\rho_2$) and the inequity in access $\log_{10}(\rho_1/\rho_2)$, expressed in favor of HI countries. Clearly, these parameters highly influence the course of the Covid-19 pandemic.

In the hypothetical scenarios, the control variable could assume whichever value. However, for the proposed eGEA scenario that we imagine feasible, we assume to reach 1% of the population injected per day ($\rho_1 = 0.01$, a peak value already realized with the current facilities), in HI countries. We assume that MLI countries can keep half of the rollout capacity of rich countries ($\rho_2 = 0.005$ a peak value already realized with the current facilities)). Note that this global daily access to vaccines ($(N_1\rho_1 + N_2\rho_2)/W = 0.0058$) has been reached end even surpassed during the real vaccination campaign in specific days: the challenge is to

constantly keep it..

S2.7 Acquisition (ν) and loss (ω) of virus resistance

We assume that vaccinated susceptibles develop full protection in about two weeks from each dose inoculation ($1/\nu = 14$ days [36]). Regarding the duration of the protection, while there is evidence that antibody levels decline substantially within a few months from infection—asymptofaimatic or mildly symptomatic subjects with fewer antibodies [67, 68]—vaccines are so far guaranteed to give protection for six months [37, 38]. At the qualitative level of this study, we optimistically assume that virus resistance wanes on average in 6 months after each dose or after recovery ($\omega = 1/180\,\mathrm{day}^{-1}$, eqs. (4a,n,o)).

S2.8 Mobility probability C

Mobility between rich and poor countries is assumed low and symmetric (for simplicity, to avoid the consideration of migration flows). For all mobile classes $Y \in \mathcal{M}$, we estimate the mobility probability as the fraction of EU citizens travelling (not for business) out of Europe (and not going to the USA) at least once in a year, resulting in approximately $C = 0.0002$ (source: ec.europa.eu/eurostat/cache/digpub/eumove). This is an underestimate of the probability C_{12} for a citizen of group 1 (HI) to be in group 2 (MLI), as business trips are not counted and an average stay abroad of one day is considered. However, as we set a symmetric mobility ($C_{12}^Y = C_{21}^Y = C$), we purposely keep this underestimate and we keep it fixed, independently of the stringency index of the source and destination groups.

S2.9 ARX filter to reconstruct the SI

In this section, we report the details on the calibration of the autoregressive-exogenous (ARX) filter to reconstruct the stringency index (SI) from hospitalization data. Focusing on a collection of 19 high-income nations, we take daily hospitalization data of each nation (fraction of the country population in hospitals with confirmed Covid-19 infection, up to September 2021) as the filter input and the corresponding daily SI in the country as output (see the caption of Table S3 for the list of countries). We treated each national input-output series as an independent experiment and we used the Matlab Identification Toolbox to identify the ARX filter from the so-obtained multi-experiment dataset.

The model that minimizes the fit error reported by the Matlab Identification Toolbox makes use of 80 autoregressive daily steps and a weighted moving average on the last 10 days of hospitalization data, achieving a fit of 46% among the single national datasets. Indeed, two-three months is the typical duration of an infection wave, during which the SI is reasonably influenced by its own previous values, while 10 days of hospitalization data represents a typical monitoring window used by our policy-makers. The model is the

following:

$$\hat{SI}(t) = \sum_{k=1}^{80} \alpha_k \hat{SI}(t-k) + \sum_{k=1}^{10} \beta_k H(t-k)/N, \tag{S1}$$

where $\hat{SI}(t)$ is the SI reconstructed estimate at day t in the considered country (or average SI across countries), $H(t)$ is the number of hospitalized subjects at the same day, and N is the population size. The coefficients α_i and β_i are reported in Table S3.

w	α_{1+7w}	α_{2+7w}	α_{3+7w}	α_{4+7w}	α_{5+7w}	α_{6+7w}	α_{7+7w}
0	-2.164	2.157	-1.879	1.011	0.165	-0.577	0.252
1	-0.254	0.669	-0.484	0.477	-1.231	1.340	-1.093
2	1.299	-1.346	1.235	-1.311	1.462	-0.727	-0.217
3	0.441	-0.513	0.855	-0.755	0.201	-0.208	0.620
4	-0.682	0.906	-1.294	0.674	0.017	0.151	-0.347
5	0.090	-0.053	0.196	0.171	-0.508	0.150	-0.147
6	0.565	-0.328	0.272	-0.839	1.087	-0.705	0.253
7	-0.158	0.028	-0.139	0.784	-0.747	0.175	-0.134
8	0.121	0.405	-0.898	0.731	-0.599	0.828	-0.551
9	0.502	-0.864	0.656	-0.174	0.286	-0.675	0.525
10	-0.516	0.495	0.275	-1.005	1.249	-1.128	0.663
11	-0.023	-0.380	0.217				

w	β_{1+7w}	β_{2+7w}	β_{3+7w}	β_{4+7w}	β_{5+7w}	β_{6+7w}	β_{7+7w}
0	537.6	364.3	213.3	24.5	-310.7	-658.9	-960.3
1	-1276.1	231.8	1835.6				

Table S3: Estimated coefficients α_i and β_i of the ARX filter eq. (S1) (grouped weekly over the autoregressive and moving average horizons of 80 and 10 days, respectively). The data used for the fitting comes from 19 countries (ISO codes): AUS, CAN, CZE, EST, FRA, HRV, HUN, IRL, ISR, ITA, LUX, NLD, PRT, SVK, SVN, SWE, GBR, and USA. Single-nation R^2 performance of model (S1): 0.71, 0.55, 0.62, 0.74, 0.61, 0.52, 0.70, 0.61, 0.56, 0.38, 0.54, 0.58, 0.32, 0.53, 0.55, 0.64, 0.57, 0.44, and 0.64.

Using the model on the aggregated data from the 19 considered nations (input signal: daily average of hospitalized population fractions; output signal: daily average SI), we obtain the result shown in the left panel of Fig.S6, where the standard deviation of the estimation error is 0.92 and the R^2 of the fitting is 0.99. The auto-correlation and cross-correlation of the residuals are reported in the right panel, showing that the information carried by the input is fully exploited, as well as most of the information carried by the past values of the SI reconstruction (some information is left in the last 10 days of reconstruction). This is due to the intrinsic nature of the SI, since containment measures are not rediscussed daily, but rather on a weekly or longer basis.

We finally check if the SI evolves similarly in middle-income nations. We tested the ARX model (S1) on 20 middle-income nations, obtaining obtain the result shown in Fig.S7, a fit of 51%. The standard deviation of the estimation error is 4.24 and the R^2 of the fitting is 0.92. This result suggests a similar decision-making process in the two macro-groups of nations (assuming, in the absence of reliable data, that

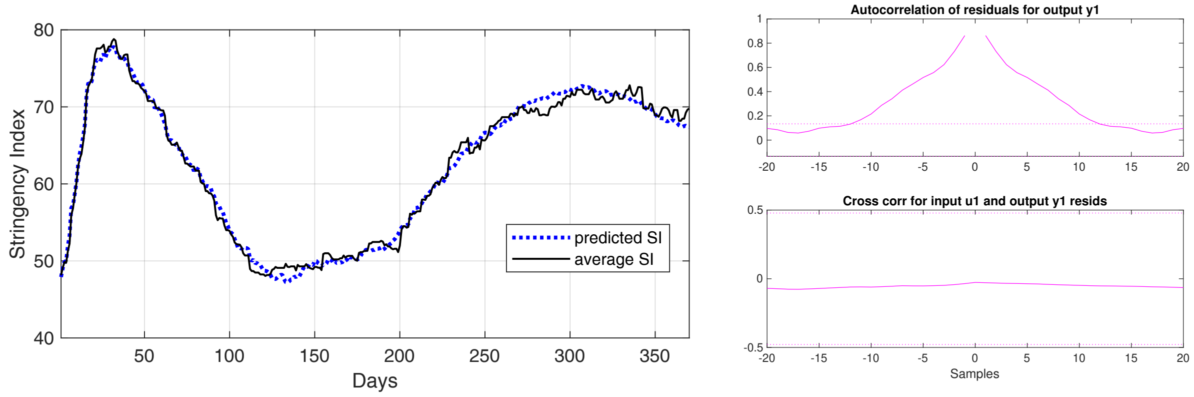

Figure S6: Left panel: Comparison between the averaged SI index available daily for high-income countries (black line, see Table S3 for the list of the 19 considered nations) and the reconstruction provided by filter (S1) fed by the average, over the same countries, of the population fractions of hospitalized subjects (blue dotted line). Right panel: Auto-correlation of the model residuals and their cross-correlation with the input signal.

low-income nations behave similarly to middle-income ones). It also provides a validation of the ARX filter itself.

We therefore implement two identical copies of filter (S1) in our model, one reconstructing the SI for HI countries and one for MLI ones. Taking the reconstructed value as the real one, we can formally remove the 'cap' from the filter output and add to our model the following two equations:

$$SI_1(t) = \sum_{k=1}^{80} \alpha_k SI_1(t-k) + \sum_{k=1}^{10} \beta_k H_1(t-k)/N_1, \quad \text{(S2)}$$

$$SI_2(t) = \sum_{k=1}^{80} \alpha_k SI_2(t-k) + \sum_{k=1}^{10} \beta_k H_2(t-k)/N_2. \quad \text{(S3)}$$

We note, however, that hospitalization flows at the beginning of the simulation do not give rise to the highest stringency observed during the early stage of the pandemic. This is due to two concomitant factors: first, the hospitalized fraction of isolated symptomatics is estimated from data averaged across 2020-22 and is much lower than in the early stage (see SI Sect. S2.3); second, the initial fear for an unknown disease contributed to the highest stringency—with a worldwide nearly synchronous lockdown. To compensate for this discrepancy between the observed and simulated stringency, we force the highest stringency level $SL_i = 5$ from day 46 ($t = 45$) for a period of 60 days, before and after which we use the stringency index provided by the filter.

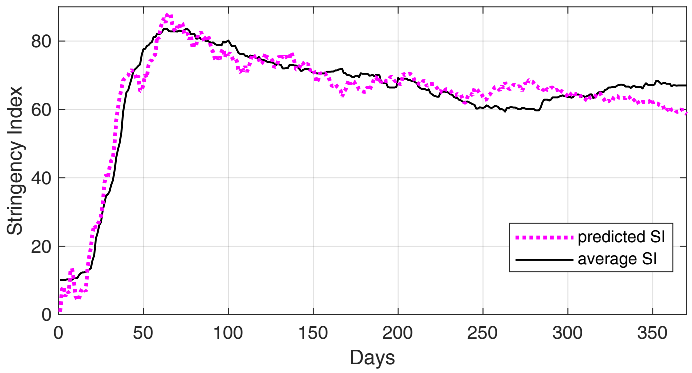

Figure S7: Comparison among the aggregated data of middle-income nations as real output (black line) and the model output (pink dotted line) using their aggregated hospitalized as input. The data used for the fitting comes from 20 nations, the stringency index source is `ourworldindata.org` [4] while the hospitalization data source is `covid19.healthdata.org` (ISO codes): ALB, DZA, ARG, AZE, BRB, BLR, BIH, BRA, BGR, CHN, CRI, DOM, ECU, GAB, IDN, IRQ, LBN, LBY, MEX, PER. Single-nation R^2 performance of model (S1): 0.73, 0.528, 0.59, 0.48, 0.44, 0.63, 0.69, 0.48, 0.47, -0.49, 0.46, 0.55, 0.52, 0.39, -0.06, 0.23, 0.47, 0.52, 0.58, and 0.65.

S3 Supplementary figures and tables

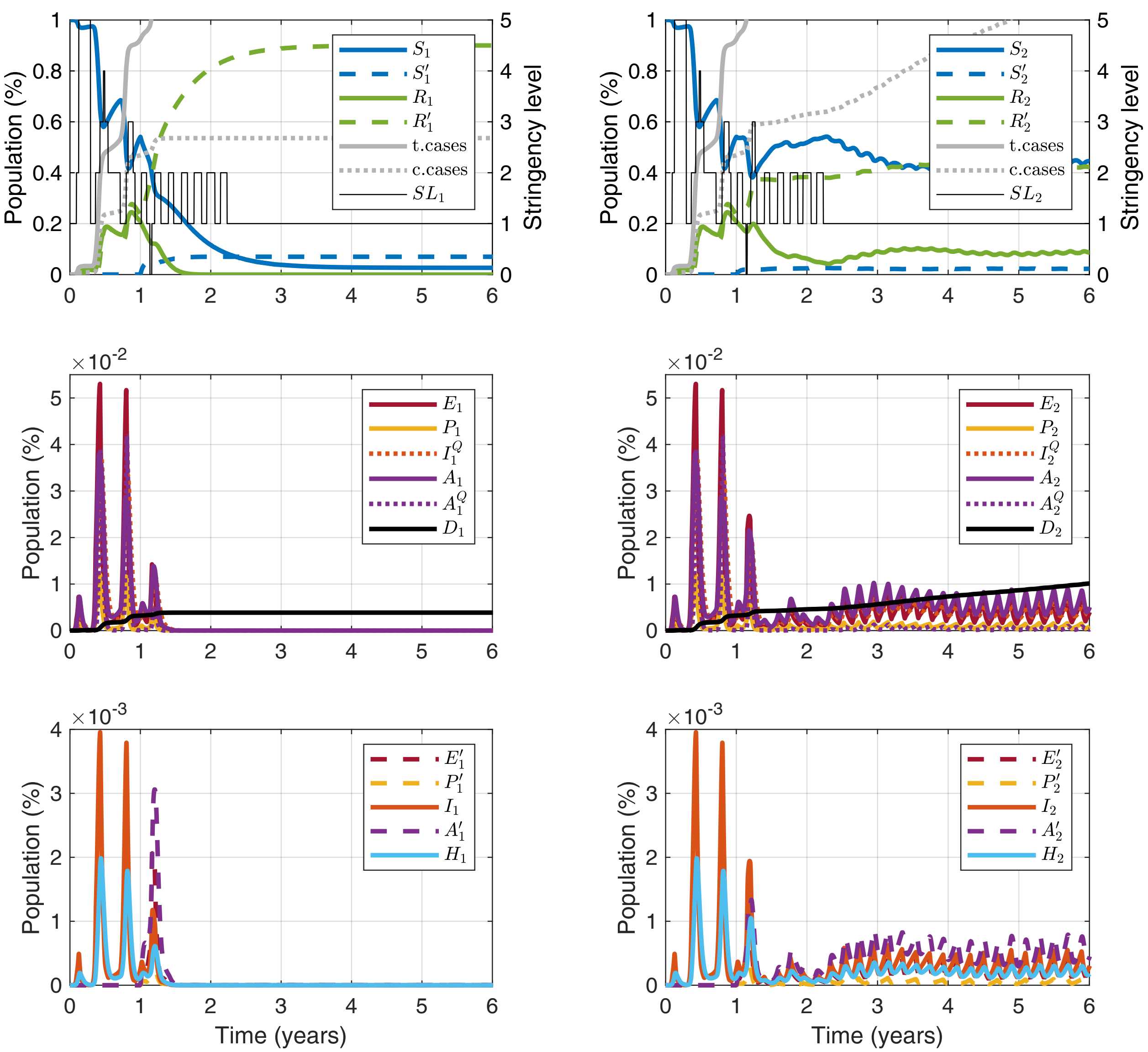

Figure S8: Model simulation of the status quo (left/right: HI/MLI countries).

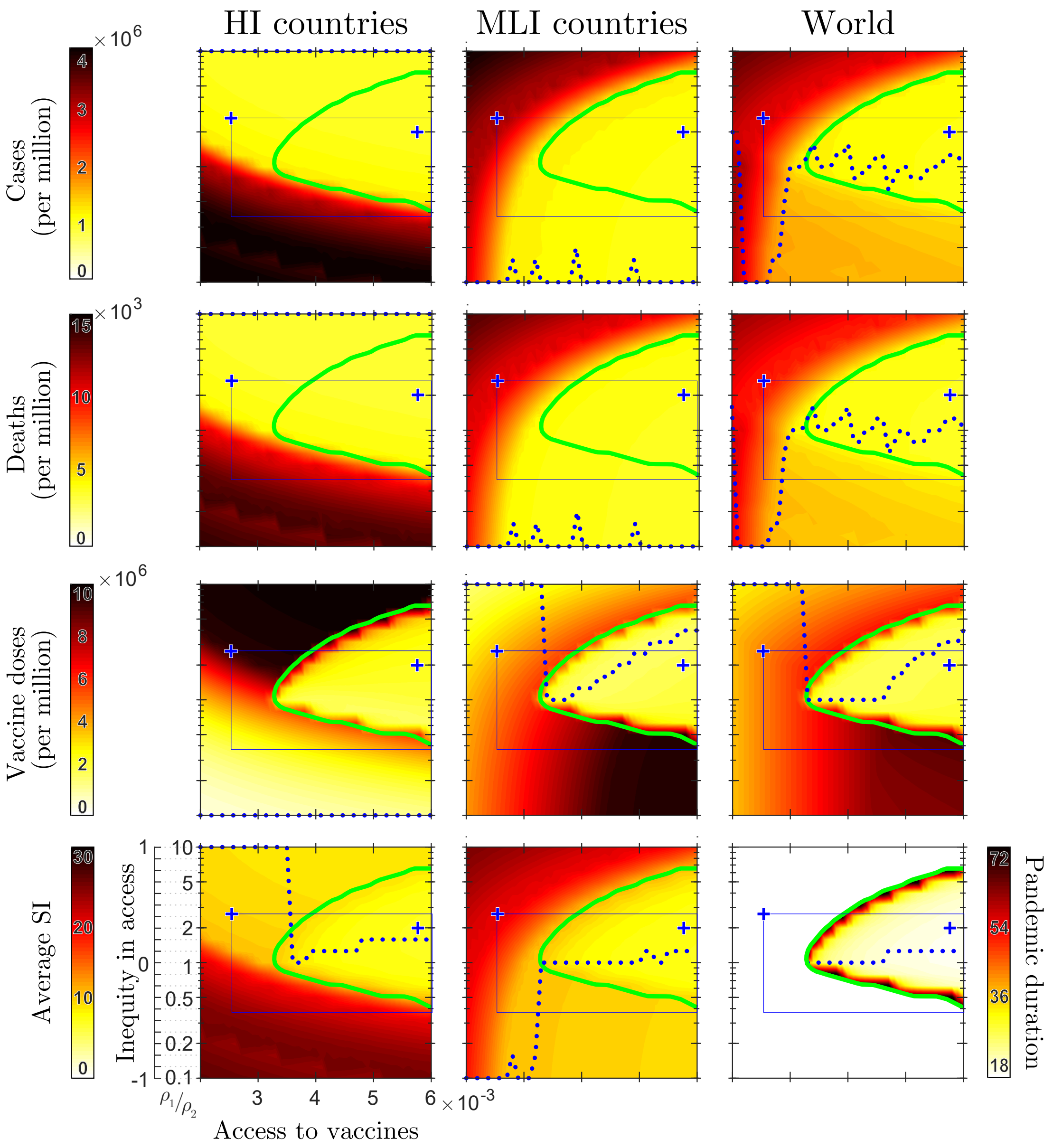

Figure S9: Analysis of the main pandemic metrics with respect to different vaccination strategies. In each panel, the top-left blue marker indicates the status quo, and is the top-left vertex of the rectangle that encapsulates all the eGEA vaccination strategies, while the other marker indicates the eGEA scenario presented in the paper. The dotted blue line shows the inequity that minimizes the cases (in HI, MLI countries and worldwide) at the specified value of access to vaccines. Vaccination strategies able to stop the pandemic before 6 years are encapsulated by the green line.

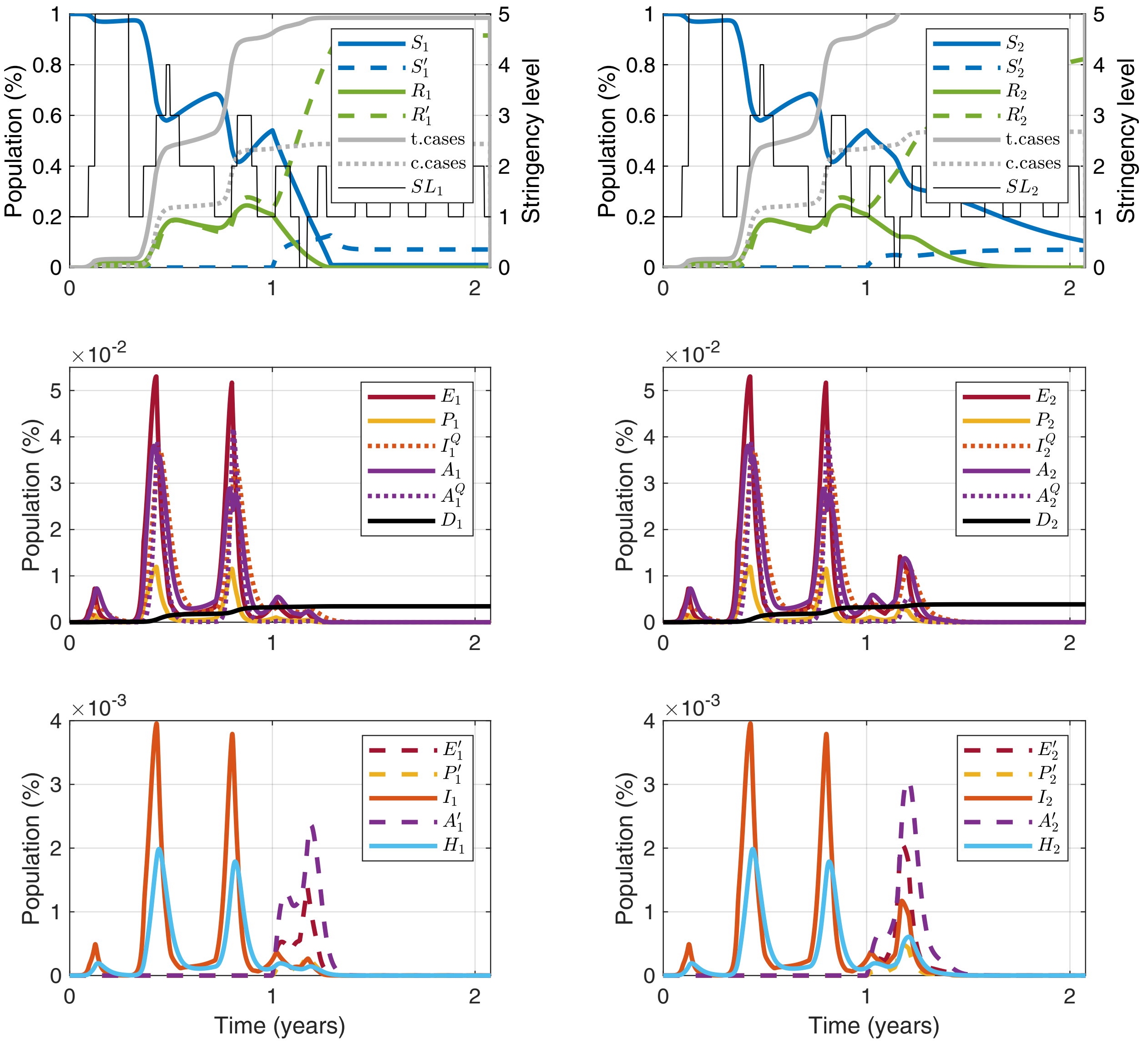

Figure S10: Model simulation of the selected eGEA scenario (left/right: HI/MLI countries). The pandemic stops in about a year of vaccination. Note that the time horizon of five years of vaccination is long enough to appreciate substantial differences between this eGEA scenario and the status quo reported in Figure S8. Most remarkably, the pandemic terminates in slightly more than a year of vaccination, while the pandemic stabilizes in an endemic regime with moderate waves in poor countries in the status quo.

Metric	status quo			GEA			$\Delta_{\%}$		
	HI	MLI	**World**	HI	MLI	**World**	HI	MLI	**World**
Pandemic length (months)	–	–	**72**	–	–	**24.2**	–	–	**-66.4%**
Total cases (M)	1417	20814	**22231**	1333.7	7002	**8335.7**	-5.8 %	-66.4%	**-62.4%**
confirmed (M)	686.1	7735	**8421.1**	648.95	3407.2	**4056.14**	-5.4 %	-56%	**-51.8 %**
% asymptomatics	48.33	32.9	**35.4**	48.62	48.62	**48.62**	6.1%	47.8%	**37.3%**
% hospitalized	3.1	4.03	**3.9**	3.1	3.1	**3.1**	0%	-23.1%	**-20.5%**
average critical h occupancy (d/y)	49	272	–	44.83	44.83	–	-8.5%	-83.5 %	–
Deaths (M)	4.9	69.2	**74.1**	4.67	24.51	**29.18**	-4.7 %	-64.5%	**-60,6 %**
Vaccines (B)	11.7	24.5	**36.2**	2.74	14.40	**17.14**	-76.6 %	-42%	**-52,6%**
Average Stringency Index	10.4	20.5	–	7.5	7.5	–	-27,8%	-63.4%	–

Table S4: Pandemic metrics in the status quo and in the GEA scenario with access to vaccines as in the vaccination-intensive eGEA scenario considered in Tab. 1 (scaled to a world population W of 8 billion people; in the GEA scenario, averaged quantities are computed by averaging a zero value after the pandemic end and up to the 6-yr-horizon of the simulation).

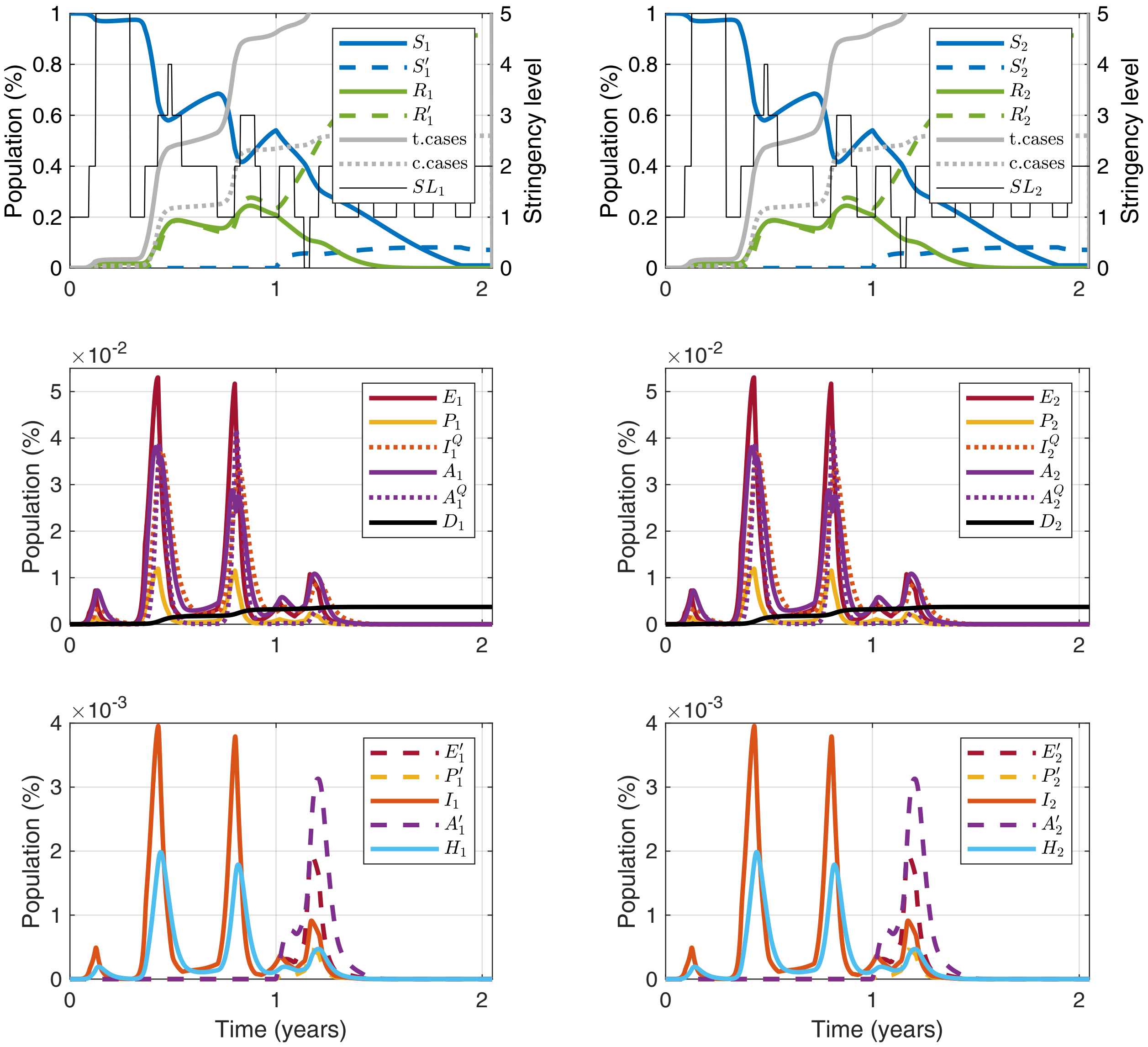

Figure S11: Model simulation of the GEA scenario ($\rho_1 = \rho_2$) with the proposed hypothetical access to vaccines presented in the paper ($(N_1\rho_1 + N_2\rho_2)/W = 0.0058$) (left/right: HI/MLI countries). The pandemic stops in about a year of vaccination.

S4 Sensitivity analysis

In what follows, we present the sensitivity analysis in absolute terms, separately for HI and MLI countries. We see that, though contact tracing and mobility do not play a crucial role (up to doubling and halving their intensity), the incidence of symptomatic cases is more critical. Specifically, a higher incidence induces less overall cases in both scenarios, because symptomatic cases are quickly isolated, but more hospitalization and deaths. It also helps in stopping the pandemic earlier in the eGEA scenario, but only up to a small increase. If the incidence is doubled, for example, the pandemic is longer than it is for a 50% increase, probably because the reduction of cases limits the spread of the protection against the virus. The same trend is consequently observed in the required vaccine doses.

Finally, we present the sensitivity analysis with respect to the average duration $\omega^{-}1$ (waning time) of vaccination-acquired or infection-acquired immunity. The effect of this parameter is crucial, giving a more severe pandemic the shorter is the duration is the waning time. Note however that the main feature of our proposed eGEA vaccination strategy, i.e., the fact that it is able to stop the pandemic in about a year of vaccination, is robust with respect to $\omega \in (\frac{1}{360}, \frac{1}{90})$, meaning with respect to losing immunity on average in a range between 3 months and 1 year.

Overall, the sensitivity analysis confirms the robustness of our model calibration. The specific effects are however not all intuitive, essentially because of the intrinsic nonlinearity of the relations. This is indeed the added value of a model-informed approach.

			probability of being symptomatic σ											
			/2	/1.5	/1.25	/1.1	/1.05	/1.03	×1.03	×1.05	×1.1	×1.25	×1.5	×2
pandemic length (days)	world	status-quo	2190	2190	2190	2190	2190	2190	2190	2190	2190	2190	2190	2190
		eGEA	805	808	783	770	763	760	754	751	748	739	737	741
cases (B)	HI	status-quo	1.62	1.51	1.43	1.41	1.42	1.42	1.42	1.42	1.42	1.40	1.37	1.24
		eGEA	1.49	1.40	1.32	1.29	1.28	1.27	1.25	1.25	1.24	1.19	1.14	1.00
	MLI	status-quo	29.14	26.68	25.24	22.60	21.78	21.25	20.45	20.23	19.71	18.31	16.46	14.10
		eGEA	8.51	7.93	7.48	7.40	7.43	7.45	7.44	7.47	7.44	7.33	7.16	6.51
deaths (M)	HI	status-quo	2.74	3.46	3.95	4.47	4.72	4.83	5.14	5.27	5.50	6.20	7.32	8.93
		eGEA	2.55	3.23	3.68	4.11	4.25	4.32	4.52	4.62	4.79	5.26	6.09	7.19
	MLI	status-quo	45.79	57.66	66.51	68.23	68.93	68.52	70.20	71.06	72.75	77.60	84.95	98.20
		eGEA	14.38	18.15	20.72	23.47	24.76	25.33	26.95	27.62	28.86	32.55	38.37	46.79
vaccines (B)	HI	status-quo	11.68	11.68	11.68	11.68	11.68	11.68	11.68	11.68	11.68	11.68	11.68	11.68
		eGEA	3.57	3.57	3.40	3.31	3.27	3.24	3.20	3.18	3.16	3.09	3.07	3.08
	MLI	status-quo	24.54	24.54	24.54	24.54	24.54	24.54	24.54	24.54	24.54	24.54	24.54	24.54
		eGEA	14.82	14.92	14.08	13.64	13.41	13.31	13.10	13.00	12.90	12.60	12.53	12.67
average SI	HI	status-quo	7.40	8.38	9.04	9.74	10.08	10.22	10.64	10.82	11.14	12.09	13.61	15.84
		eGEA	6.00	6.71	7.01	7.34	7.44	7.48	7.63	7.70	7.82	8.19	8.91	10.04
	MLI	status-quo	15.48	17.90	19.70	20.15	20.46	20.41	20.76	20.90	21.26	22.26	23.88	26.97
		eGEA	6.14	6.88	7.18	7.55	7.69	7.75	7.95	8.04	8.19	8.65	9.49	10.84

			contact tracing rate a_0											
			/2	/1.5	/1.25	/1.1	/1.05	/1.03	×1.03	×1.05	×1.1	×1.25	×1.5	×2
pandemic length (days)	world	status-quo	2190	2190	2190	2190	2190	2190	2190	2190	2190	2190	2190	2190
		eGEA	765	761	759	757	757	758	758	758	756	759	755	756
cases (B)	HI	status-quo	1.46	1.45	1.44	1.43	1.43	1.42	1.41	1.41	1.42	1.40	1.39	1.35
		eGEA	1.29	1.27	1.27	1.26	1.26	1.26	1.26	1.26	1.26	1.25	1.25	1.23
	MLI	status-quo	21.11	21.02	21.06	20.88	20.84	20.84	20.81	20.80	21.15	20.74	20.63	20.68
		eGEA	7.66	7.60	7.55	7.51	7.49	7.45	7.42	7.42	7.44	7.32	7.28	7.10
deaths (M)	HI	status-quo	5.13	5.09	5.05	5.03	5.01	4.98	4.96	4.96	4.98	4.88	4.86	4.73
		eGEA	4.52	4.48	4.45	4.43	4.43	4.42	4.41	4.41	4.40	4.38	4.36	4.32
	MLI	status-quo	70.37	70.07	70.14	69.55	69.42	69.29	69.17	69.19	70.54	68.80	68.41	68.58
		eGEA	26.90	26.69	26.50	26.35	26.26	26.11	26.01	26.01	26.10	25.61	25.48	24.83
vaccines (B)	HI	status-quo	11.68	11.68	11.68	11.68	11.68	11.68	11.68	11.68	11.68	11.68	11.68	11.68
		eGEA	3.31	3.27	3.25	3.23	3.22	3.23	3.23	3.23	3.21	3.23	3.19	3.18
	MLI	status-quo	24.54	24.54	24.54	24.54	24.54	24.54	24.54	24.54	24.54	24.54	24.54	24.54
		eGEA	13.47	13.34	13.27	13.20	13.20	13.24	13.24	13.24	13.17	13.27	13.14	13.17
average SI	HI	status-quo	10.64	10.58	10.53	10.50	10.47	10.43	10.41	10.41	10.43	10.31	10.27	10.09
		eGEA	7.70	7.64	7.60	7.58	7.57	7.56	7.55	7.55	7.53	7.53	7.49	7.45
	MLI	status-quo	20.82	20.72	20.75	20.62	20.57	20.58	20.55	20.56	20.75	20.51	20.41	20.41
		eGEA	8.03	7.97	7.92	7.89	7.88	7.86	7.85	7.85	7.84	7.80	7.75	7.67

			mobility probability $C_{i,j}$ $(i \neq j)$											
			/2	/1.5	/1.25	/1.1	/1.05	/1.03	×1.03	×1.05	×1.1	×1.25	×1.5	×2
pandemic length (days)	world	status-quo	2190	2190	2190	2190	2190	2190	2190	2190	2190	2190	2190	2190
		eGEA	758	758	758	758	758	758	758	758	758	758	758	758
cases (B)	HI	status-quo	1.42	1.42	1.42	1.42	1.42	1.42	1.42	1.42	1.42	1.42	1.42	1.42
		eGEA	1.26	1.26	1.26	1.26	1.26	1.26	1.26	1.26	1.26	1.26	1.26	1.26
	MLI	status-quo	20.84	20.86	20.87	20.82	20.82	20.82	20.81	20.81	20.81	20.81	20.81	21.39
		eGEA	7.43	7.43	7.43	7.43	7.43	7.43	7.43	7.43	7.43	7.43	7.43	7.43
deaths (M)	HI	status-quo	4.97	4.97	4.97	4.97	4.97	4.97	4.97	4.97	4.97	4.97	4.97	4.98
		eGEA	4.42	4.42	4.42	4.42	4.42	4.42	4.42	4.42	4.42	4.42	4.42	4.42
	MLI	status-quo	69.32	69.32	69.37	69.17	69.17	69.17	69.17	69.17	69.16	69.16	69.15	71.68
		eGEA	26.07	26.07	26.07	26.06	26.06	26.06	26.06	26.06	26.06	26.06	26.06	26.06
vaccines (B)	HI	status-quo	11.68	11.68	11.68	11.68	11.68	11.68	11.68	11.68	11.68	11.68	11.68	11.68
		eGEA	3.23	3.23	3.23	3.23	3.23	3.23	3.23	3.23	3.23	3.23	3.23	3.23
	MLI	status-quo	24.54	24.54	24.54	24.54	24.54	24.54	24.54	24.54	24.54	24.54	24.54	24.54
		eGEA	13.24	13.24	13.24	13.24	13.24	13.24	13.24	13.24	13.24	13.24	13.24	13.24
average SI	HI	status-quo	10.42	10.42	10.42	10.42	10.42	10.42	10.42	10.42	10.42	10.42	10.43	10.43
		eGEA	7.56	7.56	7.56	7.56	7.56	7.56	7.56	7.56	7.56	7.56	7.56	7.56
	MLI	status-quo	20.60	20.59	20.60	20.55	20.55	20.55	20.55	20.55	20.55	20.54	20.54	20.83
		eGEA	7.86	7.86	7.86	7.86	7.86	7.86	7.86	7.86	7.86	7.86	7.86	7.86

			protection waning rate (ω)											
			/2	/1.5	/1.25	/1.1	/1.05	/1.03	×1.03	×1.05	×1.1	×1.25	×1.5	×2
pandemic length (days)	world	status-quo	1235	2190	2190	2190	2190	2190	2190	2190	2190	2190	2190	2190
		eGEA	705	730	744	751	754	755	760	760	770	798	960	2190
cases (B)	HI	status-quo	1.08	1.15	1.24	1.34	1.38	1.40	1.45	1.47	1.50	1.60	1.75	4.21
		eGEA	1.07	1.12	1.17	1.22	1.24	1.25	1.28	1.29	1.31	1.41	1.53	1.72
	MLI	status-quo	6.17	9.96	16.15	20.39	20.61	20.92	21.27	21.64	22.51	24.91	27.94	33.68
		eGEA	5.68	6.03	6.49	7.05	7.24	7.34	7.59	7.72	7.85	8.36	8.91	21.66
deaths (M)	HI	status-quo	3.78	4.01	4.31	4.70	4.83	4.91	5.08	5.17	5.27	5.64	6.16	14.20
		eGEA	3.73	3.91	4.09	4.26	4.33	4.37	4.48	4.52	4.61	4.94	5.39	6.09
	MLI	status-quo	21.28	32.77	52.26	67.69	68.83	70.06	70.94	72.29	75.31	83.83	95.32	116.08
		eGEA	19.85	21.07	22.63	24.66	25.34	25.73	26.65	27.12	27.59	29.48	31.46	73.25
vaccines (B)	HI	status-quo	3.63	8.67	10.04	11.09	11.47	11.61	11.69	11.69	11.69	11.69	11.69	11.69
		eGEA	1.99	2.41	2.75	3.01	3.11	3.15	3.30	3.34	3.51	4.03	6.05	22.13
	MLI	status-quo	11.71	24.54	24.54	24.54	24.54	24.54	24.54	24.54	24.54	24.54	24.54	24.54
		eGEA	9.60	11.34	12.55	13.00	13.10	13.14	13.31	13.31	13.64	14.58	20.03	61.35
average SI	HI	status-quo	8.53	9.05	9.51	10.06	10.23	10.33	10.57	10.69	10.82	11.31	12.03	21.95
		eGEA	6.50	6.86	7.16	7.38	7.46	7.50	7.63	7.66	7.80	8.32	9.89	11.90
	MLI	status-quo	8.97	12.33	16.92	19.93	20.33	20.54	20.91	21.18	21.86	23.72	26.36	30.47
		eGEA	6.53	6.93	7.29	7.62	7.73	7.79	7.95	8.01	8.15	8.73	10.48	21.62

S4.1 Vaccine efficacy

To relax the hypothesis of 100% vaccine efficacy, we need to consider that only a fraction e of susceptible subjects develop resistance against the virus, where the new parameter e is indeed the vaccine efficacy. For modeling purposes, we assume that vaccinations on recovered resistant subjects (compartment R) have 100% efficacy, thus renewing their protection (flow $R \to R'$), and that people on which the vaccine has been ineffective, face the same infection force of susceptibles. These latter subjects are unaware of their missing protection, so that they remain ineligible for a new vaccination for the average time $\omega^{-}1$ of their presumed protection. Under such assumptions, we add a new compartment S'' for these latter subjects and consistently modify model ODEs (4) as follows:

$$\dot{S}_i = -\lambda_i^S S_i - V_i^S + \omega(R_i + R_i' + S_i'') \tag{4a'}$$

$$\dot{S}_i' = -\lambda_i^{S'} S_i' + eV_i^S - \nu S_i' \tag{4b'}$$

$$\dot{S}_i'' = -\lambda_i^{S''} S_i'' + (1-e)V_i^S - \omega S_i'' \tag{4p}$$

$$\dot{E}_i' = \lambda_i^{S'} S_i' + \lambda_i^{S''} S_i'' + V_i^E - \delta_E E_i' \tag{4d'}$$

In the following table, the parameter e is reduced to analyze the sensitivity of our simulations of the status quo and eGEA scenarios with respect to the vaccine efficacy ($e = 1$ in the original model).

			vaccine efficacy (e)					
			/2	/1.5	/1.25	/1.1	/1.05	/1.03
pandemic length (days)	world	status-quo	2190	2190	2190	2190	2190	2190
		eGEA	2190	2190	2190	814	779	769
cases (B)	HI	status-quo	4.19	3.61	2.28	1.45	1.44	1.43
		eGEA	3.75	2.11	1.32	1.28	1.27	1.27
	MLI	status-quo	26.57	24.99	23.42	21.98	21.38	21.13
		eGEA	21.95	18.87	11.24	7.59	7.52	7.49
deaths (M)	HI	status-quo	13.99	11.97	7.54	5.08	5.04	5.01
		eGEA	12.49	6.96	4.61	4.48	4.45	4.43
	MLI	status-quo	89.76	84.28	78.56	73.36	71.18	70.32
		eGEA	73.32	62.69	37.45	26.62	26.38	26.25
vaccines (B)	HI	status-quo	11.69	11.69	11.69	11.69	11.69	11.69
		eGEA	23.37	23.37	23.37	5.76	5.31	5.18
	MLI	status-quo	24.54	24.54	24.54	24.54	24.54	24.54
		eGEA	61.35	61.35	61.35	15.12	13.94	13.61
average SI	HI	status-quo	21.40	18.83	13.79	10.57	10.52	10.48
		eGEA	19.36	13.00	9.92	8.07	7.73	7.65
	MLI	status-quo	24.89	23.71	22.54	21.44	20.98	20.80
		eGEA	21.38	18.77	13.05	8.45	8.05	7.96

Interestingly, note that the change in the pandemic length (for the eGEA scenario) has similar behavior to the one obtained in the sensitivity analysis of the protection waning rate ω for small parameter perturbations (compare the /1.03, /1.05, and /1.1 columns of this table with the $\times$1.03, $\times$1.05, and $\times$1.1 columns in the table of the sensitivity analysis for the protection waning rate ω). In other words, if less effective vaccines are used either because the protection time ω^{-1} is reduced (but all the vaccinated people become protected) or because their efficacy e is reduced (but protected people remain resistant for six months on average), the model simulations are analogous, both in terms of pandemic length and in the socio-economic indicators (i.e., case, deaths, vaccines doses, and average SI). Note however that, for intermediate perturbations (1.25 and 1.5), the model simulations foresee a big difference in the eGEA scenario: the pandemic stops perturbing the protection waning rate ω, but not perturbing the vaccine efficacy e. In other words, the sensitivity with respect to e is more critical with respect to the one with respect to ω.

This result can be intuitively explained. Indeed, by reducing the vaccine efficacy, we introduce in the model subjects who are susceptible to the infection while being not eligible for a new vaccination for an average time ω^{-1}. During this time, they fuel the epidemic, independently of the vaccination rate. Differently, by increasing the waning rate ω, people lose the acquired protection in a shorter time, but as soon as they lose it they are eligible for a new vaccination. The effect on the epidemic dynamics is therefore mitigated by an intense vaccination, as that proposed in our eGEA scenario.

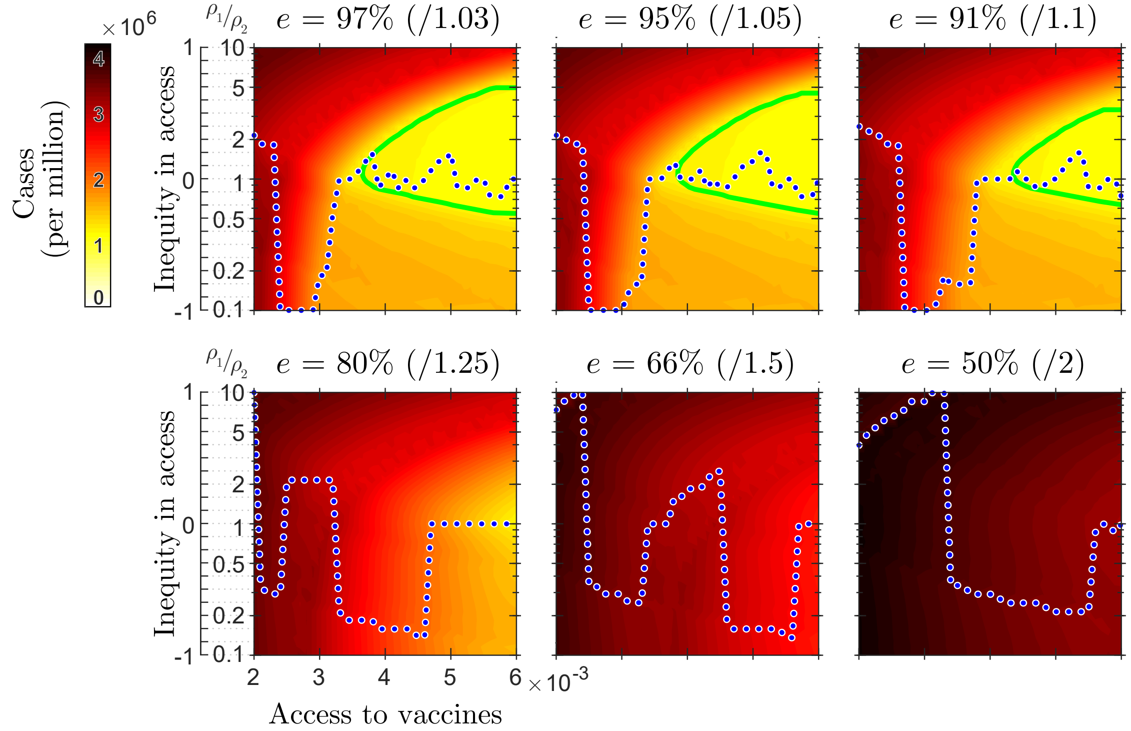

Figure S12: Analysis of the total cases with respect to different vaccination strategies for different vaccine efficacy e. The dotted blue line shows the inequity that minimizes the cases at the specified value of access to vaccines. Vaccination strategies able to stop the pandemic before 6 years are encapsulated by the green line.

We now consider the effect of a reduced vaccine efficacy on all possible vaccination strategies, defined by our two control parameters: the global access to vaccines and inequity in access between HI and MLI countries. Figure S12 reports the total cases worldwide for the same reduced values of vaccine efficacy e considered so far. As the vaccine efficacy gets smaller, it is no longer true that an intense vaccination is able to stop the pandemic. This result holds true only for $e > 65\%$ (the first five panels of the figure, even if not visible in the fourth and the fifth panels). Under this condition, the minimum access granting the pandemic end is always obtained in a GEA scenario (for $e = 80\%$ the minimum access is 0.65%, while for $e = 66\%$ it is 2.1%, unfeasibly larger than the extreme 0.6% used in the figure). But the optimality of the GEA strategy at global scale remains confirmed independently of the vaccine efficacy. The GEA strategy minimizes the total cases worldwide (and, consequently, all the pandemic metrics at global scale) if a sufficient access to vaccines is granted (see the blue dotted line, that stabilizes around zero-inequity in the right part of each panel).

References

[1] World Health Organization. Strategy to achieve global covid-19 vaccination by mid-2022 (2021).

[2] What is herd immunity (2023). URL https://www.webmd.com/covid/what-is-herd-immunity. Accessed on 2023/06/30.

[3] Setyowati, E. *et al.* Covid-19 and herd immunity: Evaluation of covid-19 vaccination policies in indonesia. *Res Militaris* **13**, 299 – 311 (2023).

[4] Mathieu, E. *et al.* Coronavirus pandemic (covid-19). *Our World in Data* (2020). Https://ourworldindata.org/coronavirus.

[5] Mathieu, E. *et al.* A global database of covid-19 vaccinations. *Nature Human Behaviour volume* **5**, 947—-953 (2021).

[6] Irwin, A. What it will take to vaccinate the world against covid-19 (2021). URL www.nature.com/articles/d41586-021-00727-3.

[7] Human rights council press releases. High commissioner for human rights: the failure to administer the covid-19 vaccines in a fair and equitable manner is prolonging the pandemic (10th March, 2022). URL www.ungeneva.org/en/news-media/meeting-summary/2022/03/conseil-des-droits-de-lhomme-nous-disposons-des-outils. Accessed on: 2022/10/12.

[8] Biancolella, M. *et al.* Covid-19 2022 update: transition of the pandemic to the endemic phase. *Human Genomics* **16**, 1–12 (2022).

[9] Morens, D. M., Taubenberger, J. K. & Fauci, A. S. Universal coronavirus vaccines — an urgent need. *New England Journal of Medicine* **386**, 297–299 (2022). URL https://doi.org/10.1056/NEJMp2118468. PMID: 34910863, https://doi.org/10.1056/NEJMp2118468.

[10] COVAX – working for global equitable access to covid-19 vaccines (2022). URL www.who.int/initiatives/act-accelerator/covax. Accessed on: 2022/10/12.

[11] A patent waiver on covid vaccines is right and fair. *Nature* **593**, 478–478 (2021).

[12] Amin, T. & Kesselheim, A. S. A global intellectual property waiver is still needed to address the inequities of covid-19 and future pandemic preparedness. *INQUIRY: The Journal of Health Care Organization, Provision, and Financing* **59**, 00469580221124821 (2022). URL https://doi.org/10.1177/00469580221124821. PMID: 36124939, https://doi.org/10.1177/00469580221124821.

[13] Emanuel, E. J. *et al.* Fair allocation of scarce medical resources in the time of covid-19. *New England Journal of Medicine* **382**, 2049–2055 (2020).

[14] Diekmann, O. & Heesterbeek, J. *Mathematical Epidemiology of Infectious Diseases: Model Building, Analysis and Interpretation.* Wiley Series in Mathematical & Computational Biology (Wiley, 2000). URL https://books.google.it/books?id=5VjSaAf35pMC.

[15] Brauer, F. & Castillo-Chavez, C. *Mathematical Models in Population Biology and Epidemiology.* Texts in Applied Mathematics (Springer, 2012). URL https://link.springer.com/book/10.1007/978-1-4614-1686-9#book-header.

[16] Brandeau, M. L., Zaric, G. S. & Richter, A. Resource allocation for control of infectious diseases in multiple independent populations: beyond cost-effectiveness analysis. *Journal of Health Economics* **22**, 575–598 (2003). URL https://www.sciencedirect.com/science/article/pii/S0167629603000432.

[17] E., R. R., Ramanan, L. & A., G. C. Optimal control of epidemics in metapopulations. *J. R. Soc. Interface* **6**, 1135–1144 (2009).

[18] Ndeffo Mbah, M. L. & Gilligan, C. A. Resource allocation for epidemic control in metapopulations. *PLOS ONE* **6**, 1–10 (2011). URL https://doi.org/10.1371/journal.pone.0024577.

[19] Kermack, W. O. & McKendrick, A. G. A contribution to the mathematical theory of epidemics. *Proceedings of the Royal Society of London. Series A, Containing Papers of a Mathematical and Physical Character* **115**, 700–721 (1927). URL http://www.jstor.org/stable/94815.

[20] Lin, Q. *et al.* A conceptual model for the coronavirus disease 2019 (covid-19) outbreak in wuhan, china with individual reaction and governmental action. *International Journal of Infectious Diseases* **93**, 211 – 216 (2020).

[21] Gatto, M. *et al.* Spread and dynamics of the covid-19 epidemic in italy: Effects of emergency containment measures. *Proceedings of the National Academy of Sciences* **117**, 10484–10491 (2020).

[22] Anastassopoulou, C., Russo, L., Tsakris, A. & Siettos, C. Data-based analysis, modelling and forecasting of the covid-19 outbreak. *PLOS ONE* **15**, 1–21 (2020). URL https://doi.org/10.1371/journal.pone.0230405.

[23] Della Rossa, F. *et al.* A network model of italy shows that intermittent regional strategies can alleviate the covid-19 epidemic. *Nature communications* **11**, 1–9 (2020).

[24] Casella, F. Can the COVID-19 epidemic be controlled on the basis of daily test reports? *IEEE Control Systems Letters* **5**, 1079–1084 (2021).

[25] Yang, W., Zhang, D., Peng, L., Zhuge, C. & Hong, L. Rational evaluation of various epidemic models based on the covid-19 data of china (2021). 2003.05666.

[26] Moore, S., Hill, E. M., Dyson, L., Tildesley, M. J. & Keeling, M. J. Retrospectively modeling the effects of increased global vaccine sharing on the covid-19 pandemic. *Nature Medicine* **28**, 2416 – 2423 (2022).

[27] Castonguay, F. M. *et al.* Optimal spatial evaluation of a pro rata vaccine distribution rule for covid-19. *Scientific Reports* **13** (2023).

[28] Mathieu, E. *et al.* A global database of covid-19 vaccinations. *Nature human behaviour* **5**, 947–953 (2021).

[29] Lopez Bernal, J. *et al.* Effectiveness of covid-19 vaccines against the b.1.617.2 (delta) variant. *New England Journal of Medicine* **385**, 585–594 (2021). URL https://doi.org/10.1056/NEJMoa2108891. PMID: 34289274, https://doi.org/10.1056/NEJMoa2108891.

[30] Mohammed, I. *et al.* The efficacy and effectiveness of the covid-19 vaccines in reducing infection, severity, hospitalization, and mortality: a systematic review. *Human Vaccines & Immunotherapeutics* **18**, 2027160 (2022). URL https://doi.org/10.1080/21645515.2022.2027160. PMID: 34346833, https://doi.org/10.1080/21645515.2022.2027160.

[31] Rahmani, K. *et al.* The effectiveness of covid-19 vaccines in reducing the incidence, hospitalization, and mortality from covid-19: A systematic review and meta-analysis. *Frontiers in Public Health* **10** (2022). URL https://www.frontiersin.org/articles/10.3389/fpubh.2022.873596.

[32] Chi, W.-Y. *et al.* Covid-19 vaccine update: vaccine effectiveness, sars-cov-2 variants, boosters, adverse effects, and immune correlates of protection. *Journal of Biomedical Science* **29** (2022). URL https://www.scopus.com/inward/record.uri?eid=2-s2.0-85139886836&doi=10.1186%2fs12929-022-00853-8&partnerID=40&md5=77bde74fc881a906da2ff74b7c5a3cbe.

[33] Firouzabadi, N., Ghasemiyeh, P., Moradishooli, F. & Mohammadi-Samani, S. Update on the effectiveness of covid-19 vaccines on different variants of sars-cov-2. *International Immunopharmacology* **117** (2023). URL https://www.scopus.com/inward/record.uri?eid=2-s2.0-85149823137&doi=10.1016%2fj.intimp.2023.109968&partnerID=40&md5=439cec5ae6ecb362723fe503044e595f.

[34] Hale, T. *et al.* A global panel database of pandemic policies (oxford covid-19 government response tracker). *Nature human behaviour* **5**, 529–538 (2021).

[35] Nishiura, H., Linton, N. M. & Akhmetzhanov, A. R. Serial interval of novel coronavirus (covid-19) infections. *International journal of infectious diseases* **93**, 284–286 (2020).

[36] WHO Collaborating Center for Vaccine Safey. How quickly does the vaccine work and how long does the protection last? (2023). URL www.covid19infovaccines.com/en-posts/how-quickly-does-the-vaccine-work-and-how-long-does-the-protection-last. Accessed on: 2023/09/05.

[37] Pfizer and biontech confirm high efficacy and no serious safety concerns through up to six months following second dose in updated topline analysis of landmark covid-19 vaccine study (2021). URL www.pfizer.com/news/press-release/press-release-detail/pfizer-and-biontech-confirm-high-efficacy-and-no-serious. Accessed on 2022/11/17.

[38] Moderna provides clinical and supply updates on covid-19 vaccine program ahead of 2nd annual vaccines day (2021). URL www.businesswire.com/news/home/20210413006131/en/. Accessed on 2022/11/17.

[39] Fang, Y. *et al.* Sensitivity of chest ct for covid-19: Comparison to rt-pcr. *Radiology* **296**, 200432 (2020).

[40] Ai, T. *et al.* Correlation of chest ct and rt-pcr testing for coronavirus disease 2019 (covid-19) in china: A report of 1014 cases. *Radiology* **296**, 200642 (2020).

[41] Katella, K. Comparing the covid-19 vaccines: How are they different? (2023). URL www.yalemedicine.org/news/covid-19-vaccine-comparison. Accessed on: 2023/07/06.

[42] Dunkle, L. M. *et al.* Efficacy and safety of nvx-cov2373 in adults in the united states and mexico. *New England Journal of Medicine* **386**, 531–543 (2022). URL https://doi.org/10.1056/NEJMoa2116185. PMID: 34910859, https://doi.org/10.1056/NEJMoa2116185.

[43] Constantino, A. K. Pmoderna, pfizer say updated covid vaccines were effective against highly mutated ba.2.86 variant in trials (2023). URL https://www.cnbc.com/2023/09/06/moderna-says-new-covid-vaccine-effective-against-bapoint2point86-variant.html. Accessed on 2023/09/14.

[44] Menegale, F. *et al.* Evaluation of waning of sars-cov-2 vaccine–induced immunity: A systematic review and meta-analysis. *JAMA Network Open* **6**, e2310650–e2310650 (2023).

[45] Jennings, K. How much will it cost to get a covid-19 vaccine? (2022). URL www.healthline.com/health-news/how-much-will-it-cost-to-get-a-covid-19-vaccine. Accessed on: 2022/11/17.

[46] K, K., AZ, B., YK, D. & R, R. How is covid-19 altering the manufacturing landscape? a literature review of imminent challenges and management interventions. *Ann Oper Res* 1353–1362 (2021).

[47] ST, A. *et al.* Challenges to covid-19 vaccine supply chain: Implications for sustainable development goals. *Int J Prod Econ* 1353–1362 (2021).

[48] Massinga Loembé, M. & Nkengasong, J. N. Covid-19 vaccine access in africa: Global distribution, vaccine platforms, and challenges ahead. *Immunity* **54**, 1353–1362 (2021).

[49] Machingaidze, S. & Wiysonge, C. Understanding covid-19 vaccine hesitancy. *Nature Medicine* **27**, 1338–1339 (2021).

[50] Ancona, C., Iudice, F. L., Garofalo, F. & De Lellis, P. A model-based opinion dynamics approach to tackle vaccine hesitancy. *Scientific Reports* **12**, 11835 (2022).

[51] Bubar, K. M. *et al.* Model-informed covid-19 vaccine prioritization strategies by age and serostatus. *Science* **371**, 916–921 (2021).

[52] Sanz-Leon, P. *et al.* Modelling herd immunity requirements in queensland: impact of vaccination effectiveness, hesitancy and variants of sars-cov-2. *Philosophical Transactions of the Royal Society A: Mathematical, Physical and Engineering Sciences* **380** (2022). URL `https://www.scopus.com/inward/record.uri?eid=2-s2.0-85134401309&doi=10.1098%2frsta.2021.0311&partnerID=40&md5=1d41356f936e164328b92577d8add208`.

[53] Vattiato, G. *et al.* An assessment of the potential impact of the omicron variant of sars-cov-2 in aotearoa new zealand. *Infectious Disease Modelling* **7**, 94 – 105 (2022). URL `https://www.scopus.com/inward/record.uri?eid=2-s2.0-85128200615&doi=10.1016%2fj.idm.2022.04.002&partnerID=40&md5=ea260177ce589c88565ea9b03e95514b`.

[54] MacIntyre, C. R., Costantino, V. & Trent, M. Modelling of covid-19 vaccination strategies and herd immunity, in scenarios of limited and full vaccine supply in nsw, australia. *Vaccine* **40**, 2506 – 2513 (2022). URL `https://www.scopus.com/inward/record.uri?eid=2-s2.0-85105250528&doi=10.1016%2fj.vaccine.2021.04.042&partnerID=40&md5=1aa7741d79534d2b9e1f62360dbcade7`.

[55] Morens, D. M., Folkers, G. K. & Fauci, A. S. The Concept of Classical Herd Immunity May Not Apply to COVID-19. *The Journal of Infectious Diseases* **226**, 195–198 (2022).

[56] Covid-19 nelle scuole del veneto: positivi 3.369 studenti e 263 docenti e operatori (2021). URL `www.veronasera.it/cronaca/coronavirus-covid-19-scuola-veneto-1-dicembre-2021.html`. Accessed on 2022/11/17.

[57] Green, M. S. *et al.* When is an epidemic an epidemic? *The Israel Medical Association Journal* **4**, 3–6 (2002).

[58] Centers for Disease Control and Prevention. Principles of epidemiology in public health practice. *An introduction to applied epidemiology and biostatistics, 3rd edn. Public Health Foundation, Washington, DC* (2012).

[59] Montgomery, D. C., Runger, G. C. & Hubele, N. F. *Engineering statistics* (John Wiley & Sons, 2009).

[60] Saville, D. J. Multiple comparison procedures: the practical solution. *The American Statistician* **44**, 174–180 (1990).

[61] Krzanowski, W. *Principles of multivariate analysis*, vol. 23 (OUP Oxford, 2000).

[62] Ma, Q. *et al.* Global percentage of asymptomatic sars-cov-2 infections among the tested population and individuals with confirmed covid-19 diagnosis: A systematic review and meta-analysis. *JAMA Network Open* **4**, e2137257 (2021).

[63] Hellewell, J. *et al.* Feasibility of controlling covid-19 outbreaks by isolation of cases and contacts. *The Lancet Global Health* **8**, e488–e496 (2020).

[64] Ngonghala, C. N. *et al.* Mathematical assessment of the impact of non-pharmaceutical interventions on curtailing the 2019 novel coronavirus. *Mathematical biosciences* **325**, 108364 (2020).

[65] Kucharski, A. J. *et al.* Effectiveness of isolation, testing, contact tracing, and physical distancing on reducing transmission of sars-cov-2 in different settings: a mathematical modelling study. *The Lancet Infectious Diseases* **20**, 1151–1160 (2020).

[66] Keeling, M. J., Hollingsworth, T. D. & Read, J. M. Efficacy of contact tracing for the containment of the 2019 novel coronavirus (covid-19). *J Epidemiol Community Health* **74**, 861–866 (2020).

[67] Long, Q.-X. *et al.* Clinical and immunological assessment of asymptomatic sars-cov-2 infections. *Nature medicine* **26**, 1200–1204 (2020).

[68] Ibarrondo, F. J. *et al.* Rapid decay of anti–sars-cov-2 antibodies in persons with mild covid-19. *New England Journal of Medicine* **383**, 1085–1087 (2020).
